# Space-time trends in fetal mortality in Brazil, 1996–2021

**DOI:** 10.11606/s1518-8787.2025059006194

**Published:** 2025-03-31

**Authors:** João Batista Francalino da Rocha, Italla Maria Pinheiro Bezerra, Elyecleyde Katiane da Silva Oliveira, Aline Bergamini Effgen Sena, Francisco Naildo Cardoso Leitão, Luiz Carlos de Abreu

**Affiliations:** I Faculdade de Medicina do ABC. Centro Universitário. Programa de Pós-Graduação em Ciências da Saúde. Santo André, SP, Brasil; II Escola Superior de Ciências da Santa Casa de Misericórdia de Vitória. Programa de Pós-Graduação em Políticas Públicas e Desenvolvimento Local. Vitória, ES, Brasil; III Universidade Federal do Acre. Laboratório Multidisciplinar de Estudos e Escrita Científica em Ciências da Saúde. Rio Branco, AC, Brasil; IV Universidade Federal do Espírito Santo. Departamento de Educação Integrada em Saúde. Vitória, ES, Brasil; V Universidade Federal do Acre. Programa de Pós-Graduação em Ciências da Saúde. Rio Branco, AC, Brasil; VI Universidade Federal do Espírito Santo. Programa de Pós-Graduação em Saúde Coletiva. Vitória, ES, Brasil

**Keywords:** Perinatal Mortality, Fetal Mortality, Fetal Death, Stillbirth, Perinatal Care, COVID-19, Health Inequalities

## Abstract

**OBJECTIVE:**

To evaluate the space-time trend of fetal mortality in Brazil from 1996 to 2021.

**METHODS:**

Ecological time series study with secondary data on fetal deaths at gestational age (GA) ≥ 20 weeks from the Mortality Information System (SIM) in Brazil, between 1996 and 2021, covid-19 pre-pandemic (1996–2019), and years 2020 and 2021 of the pandemic. It analyzed the fetal mortality rate (FMR) to identify the annual risk of fetal death in the specific population. The percentage change (PC), annual percentage change (APC), and average annual percentage change (AAPC) were calculated using Joinpoint regression to determine the trend patterns: increasing, decreasing, or stationary. Excel 2019, Stata, and Joinpoint Regression software were used.

**RESULTS:**

In Brazil, fetal deaths at GA ≥ 20 weeks accounted for 1.14% of births and 58% of perinatal deaths in the period analyzed. Around 93% were antepartum, 6% intrapartum, and 1% were recorded as occurring postpartum. The overall FMR for the period, considering GA ≥ 20, ≥ 22, and ≥ 28 weeks, was 11.4, 10.7, and 8.6/1,000 births, respectively. Despite the increasing trend of stillbirths in perinatal deaths, a slowdown in the reduction and an increase during the covid-19 pandemic, the FMR at the gestational ages evaluated decreased by 20%, 25%, and 41%, respectively, with a AAPC of -1.00, -1.13, and -1.89.

**CONCLUSION:**

Fetal mortality showed a long-term downward trend at the national and regional levels in Brazil, except in the Central-West region, where the trend was stationary. The covid-19 pandemic slowed down the reduction and increased the measure, however, the downward trend was not interrupted. Regionally, the highest FMRs and the smallest reductions occurred in the North, Northeast, and Central-West, showing regional inequalities.

## INTRODUCTION

Fetal mortality is one of the maternal and child health indicators widely used to analyze the living conditions of a population, since it reflects the occurrence of factors linked to pregnancy and childbirth, conditions of access to health services, and the quality of care provided to women during pregnancy^1^. It is a common adverse outcome of pregnancy, most of which can be avoided with appropriate interventions^4^, however, prevention and reduction are still invisible in programmatic actions and health monitoring reports around the world^2,4^.

Stillbirths were not counted in the Millennium Development Goals (MDGs), nor monitored by the United Nations (UN), nor computed in the Global Burden of Disease metrics, nor did they have a specific target in the Sustainable Development Goals (SDGs)^5^. However, interventions to reduce maternal and under-five mortality in the MDGs have helped to reduce fetal mortality, which has received increasing attention in global agendas and programmatic actions^6^.

In the SDGs, target 3.1 is to reduce global maternal mortality to less than 70 deaths per 1,000 live births and target 3.2 is to end preventable deaths of newborns and children under five by reducing neonatal mortality to at least 12 per 1,000 live births and under-five mortality to at least 25 per 1,000 live births by 2030. Although there is no specific target for fetal mortality, its reduction is fundamental to achieving these goals^7^.

The World Health Organization’s (WHO) Global Strategy for Women’s, Children’s and Adolescents’ Health (2016–2030) and the Every Newborn Action Plan (ENAP) led by the United Nations Children’s Fund (Unicef) and WHO include actions to prevent maternal and child mortality, including reducing fetal mortality by strengthening maternal and newborn health services^7^.

The Pan American Health Organization (PAHO) has supported countries in the region in their efforts to monitor fetal mortality, train professionals, and strengthen health information systems to monitor this indicator^8^. Many countries have incorporated targets and actions to reduce fetal mortality into their maternal and child health plans and policies, such as the National Plan for the Prevention of Maternal and Child Mortality in Brazil^6^.

Globally, there was a 35% reduction in the stillbirth rate between 2000 and 2021, among fetuses with gestational age (GA) ≥ 28 weeks, which decreased from 21.3 stillbirths per 1,000 total births in 2000 to 13.9 in 2021, with an annual reduction rate of 2.0^9,10^. Although some progress has been made, fetal mortality remains a global problem^11^ with around 2 million stillbirths per year in recent years, resulting in a tremendous loss of 53 million stillbirths since the year 2000^12^.

In Latin America and the Caribbean, estimates point to a 31% reduction in the stillbirth rate (GA ≥ 28 weeks), from 11.2 stillbirths per 1,000 total births to 7.74 in the same period^9,10^. In Brazil, the stillbirth rate at GA ≥ 28 weeks decreased from 10.12 stillbirths per 1,000 total births in 2000 to 6.98 total births in 2015^9^. When counting perinatal deaths, considering the national criterion of 22 weeks of gestation up to six days of life, 96.62% of perinatal deaths were classified as stillbirths^13^. According to the WHO criterion of 28 weeks to six days gestation, this proportion was 85.53% in the country^10^.

Brazil is a country with great socio-economic and regional inequalities, which can directly influence maternal and child health indicators, including fetal mortality. Factors such as low maternal schooling, poor housing and sanitation conditions, difficulty in accessing health services, and poor quality of prenatal care tend to be more prevalent in poorer regions, such as the North and Northeast^14^.

Around 73% of stillbirths are caused by prematurity and low birth weight (< 2,500 grams)^7^. Almost half of all stillbirths occur during labor and birth^2,4^. As such, most deaths result from problems that can be avoided with adequate maternal and fetal care, such as the prevention or treatment of infections and pregnancy complications (e.g. hypertension, diabetes, and growth retardation of the fetus before birth)^5,11^.

However, in addition, its reduction requires equitable access to quality perinatal care, maternal education, professional training and improved socioeconomic conditions^2^, and continuous monitoring and analysis of perinatal mortality data is crucial to implementing effective reduction policies^8^. The covid-19 pandemic has imposed additional challenges to this scenario for fetal health both locally and globally, which need to be quantified^15,16^.

The aim of this study was to assess the spatio-temporal trend in fetal mortality in Brazil from 1996 to 2021. This time interval includes a pre-pandemic period (1996–2019) and the years 2020 and 2021 of the covid-19 pandemic. The hypothesis was that fetal mortality, both at the national level in Brazil, and regionally in its Major Regions, would show significant long-term downward trends over time, in the pre-pandemic period, and including the covid-19 pandemic (1996–2021).

Three specific objectives were established: 1) to identify the patterns of trends in fetal mortality rates at national and regional level over the period analyzed; 2) to identify patterns and geographical variations in fetal mortality between major regions during the same period; 3) to describe the patterns of risk and trends, by demographic and obstetric factors, as well as by causes of death in fetal mortality rates at national level during the period under analysis. This data and information can support greater attention and targeted actions to reduce preventable fetal deaths more quickly and effectively.

## METHODS

### Study Design and Population

Ecological time series study of annual fetal mortality rates in Brazil between 1996 and 2021, comprising the pre-pandemic period (1996–2019) and the years 2020 and 2021 of the covid-19 pandemic. Secondary data was used regarding the registration of fetal deaths with GA ≥ 20 weeks from the Mortality Information System (SIM) of the Department of Informatics of the Unified Health System (Datasus) of the Brazilian Ministry of Health (MS)^17^.

Annual percentage and mean annual variations were estimated to determine trend patterns for absolute measures, for the number of stillbirths, and relative measures, for the proportions and rates of fetal mortality, both for Brazil and its Major Regions (North, Northeast, Southeast, South, and Central-West). For these estimates, stillbirths were considered in the strata of GA ≥ 20, ≥ 22, and ≥ 28 weeks.

Nationwide, the study population consisted of 886,878 records of fetal deaths with GA ≥ 20 weeks. At the regional level, the study population was made up of these fetal death records, according to the register by Brazilian Major Region, which totaled 886,852 cases (26 cases less than the national total), corresponding to 92.613 cases (10.44%) in the North, 285,896 cases (32.24%) in the Northeast, 350,546 cases (39.53%) in the Southeast, 97,187 cases (10.96%) in the South, and 60,610 cases (6.83%) in the Central-West during the period analyzed.

The stillbirth population at the national level was disaggregated into three gestational age strata: GA ≥ 20 weeks (100% of records); GA ≥ 22 weeks, 835,153 cases (94% of records); and GA ≥ 28 weeks, 665,287 cases (75% of records). At the regional level, it was stratified into: GA ≥ 20 weeks, according to the distribution mentioned above; and GA ≥ 22 weeks, 835,130 cases, distributed by Major Region as follows: 86,916 cases (10.41%) in the North, 270,825 cases (32.43%) in the Northeast, 327,118 cases (39.17%) in the Southeast, 92,900 cases (11.12%) in the South, and 57,371 cases (6.87%) in the Central-West.

This stratification of the stillbirth population, at national and regional levels, was performed to allow the analysis of fetal deaths: 1) regarding registration from GA ≥ 20 weeks, standardized by the Brazilian Ministry of Health with the issuance of the Death Certificate (DC) and registration in the SIM^18,19^; 2) regarding the classification of stillbirths with GA ≥ 22 weeks, recommended by the WHO and adopted in ICD-10^20^, and mandatory GA for investigation of fetal death standardized by the Ministry of Health^13^ and comparisons within the country; 3) regarding GA ≥ 28 weeks, an international classification that allows, an international classification that allows comparisons between countries and global regions.

To allow the calculate fetal mortality rates (FMR), the annual population of total births (NT) was adopted, which totaled 77,910,333 records, an annual average of 2,910,333, in the period from 1996 to 2021. This was composed of 77,075,180 records of live births extracted from the Live Birth Information System (SINASC) and records of stillborn fetal deaths. It is noteworthy that the total NT births were also structured into three strata, they were composed considering, in the sum, the number of stillbirths considering the GA: ≥ 20; ≥ 22; and ≥ 28 weeks.

### Data Collection and Procedures

Data on stillbirths were collected from SIM and live births from SINASC, tabulated using TabNet online. TabNet is a tabulation tool developed by DATASUS, the Brazilian Ministry of Health, which allows online tabulation of data and the generation of spreadsheets from the database of the Brazilian Unified Health System (SUS). It is a public domain tabulator, available at https://datasus.saude.gov.br/informacoes-de-saude-tabnet/. The data was accessed on January 7 and 8, 2022^17^.

The steps followed in extracting data on stillbirths were in TabNet, we accessed “Vital Statistics”, “Mortality - since 1996 by ICD-10”, “Fetal deaths”, coverage area “Brazil by Region and Federation Unit”. In the tabulation environment, in the “Column” field, the year of death was selected; in the “Content” field, death by residence; in the “Available periods”, the dates that matched the study; in the “Available selections” field, in the duration of pregnancy, three strata of GA were considered: ≥ 20, ≥ 22, and ≥ 28 weeks, plus the “unknown” category; finally, in the “Row” field, the variables of interest to the study were selected one at a time^17^.

To extract data on live births, the following steps were followed: in TabNet, access “Vital Statistics”, “Live births - since 1994”, select “Live births”, area of coverage “Brazil by Region and Federation Unit”. In the tabulation environment, in the “Column” field, the year of birth was selected; in the “Content” field, the birth by mother’s residence; in the “Available periods”, the dates that matched the study; and in the “Row” field, one at a time, the variables of interest to the research were selected^17^.

The outcome investigated was the trend patterns of fetal mortality rates in Brazil over time, at national and regional levels. The aim was to identify whether these rates were increasing, decreasing, or stationary. To this end, the percentage variation (PV), the annual percentage variation (APC) and the average annual percentage variation (AAPC) were estimated.

The predictor variable is “time”, expressed in calendar years, which was used to model the evolution of fetal mortality rates. Fetal mortality rates were explored by characteristics, grouped into: sociodemographic and obstetric and by chapter and list of causes of death according to the International Classification of Diseases (ICD-10).

The group of sociodemographic variables composed of: sex of the stillbirth (male and female); maternal age in years, categorized into age classes with intervals of five years each, starting at 10-14 up to 49 years and over; maternal schooling in complete years of study of the pregnant woman at the time of delivery, categorized into: none, 1-3 years, 4-7 years, 8-11 years, and 12 years or more.

The group of obstetric variables included: duration of pregnancy in weeks, categorized as < 22 weeks and, for GA ≥ 22 weeks, was distributed into categories with intervals of five weeks up to 42 weeks and more; birth weight in grams (g), categorized as < 500 g, and from 500 g onwards, distributed in intervals of 500 g up to 2,500–2,999 g, 3,000–3,999 g, and 4,000 g and over; type of pregnancy (single, double, and triple and over); and type of delivery (cesarean and vaginal).

Fetal mortality was also analyzed in relation to the place of occurrence (hospital, other health facility, home, other places such as public roads and indigenous villages); time of fetal death in relation to childbirth (before, during, and after); underlying cause of fetal death categorized by chapters and list of causes; and Major Regions: North, Northeast, Southeast, South, and Central-West of Brazil.

### Statistical Analysis

The FMRs was calculated for the strata of GA ≥ 20, ≥ 22, and ≥ 28 weeks. When calculating the FMR, considering the underreporting of fetal deaths and the precariousness of information on the length of pregnancy, the number of fetal deaths with an unknown or unfilled GA were added to the numerator and denominator. The calculation method used was direct, applying the following formula:

fetal mortality coefficient or fetal mortality rate, calculation method.


$$\mathrm{FMR}=\frac{\begin{array}{c} \text{ Number of fetal deaths (22-week gestation }\\ \text{ and over), of resident mothers } \end{array}}{\begin{array}{c} \text{ Number of total births to resident }\\ \text{ mothers (live births plus fetal deaths }\\ \text{ at }22\text{ weeks or more of gestation) } \end{array}}\times 1.000$$ (1)

The explanatory analysis of the trend patterns of the fetal mortality time series was carried out by constructing non-segmented and segmented regression models of the indices of this indicator, using the Joinpoint Regression Analysis program^21^. In other words, both the long-term trend analysis and the interrupted trend analysis were carried out, checking whether a line with one point or multiple inflection points was statistically significant, considering alpha = 0.05.

In the analysis of the interrupted (segmented) trend in the time series of the indices of the indicator analyzed, the number of inflection points to obtain the significant model was selected using the software’s default settings using the Grid Search method (which allows the inflection points to occur exactly in the years observed).

The statistical significance tests for choosing/evaluating the best regression model were based on the Monte Carlo permutation method^22,23^, especially useful considering the data set analyzed, where the assumptions of parametric tests (such as normality or homoscedasticity) are violated and the theoretical distribution of the statistical test is difficult to calculate^22,23^.

To analyze the trend patterns in the Joinpoint Regression software, the modeling defined the absolute frequency and proportion of stillbirths and the FMR by category of the study variables as outcomes, and the years of occurrence of these indicators as the predictor variable.

The Joinpoint Regression Analysis^21^ software was configured to calculate the AAPC for the following periods: 1996–2019 - prior to the covid-19 pandemic; and 1996–2021 - interval including the years of the covid-19 pandemic. Additionally, in cases where the rows showed one or more inflection points, the APC was calculated for each segment^22,23^.

In the case of a trend without a segment, the AAPC is equal to the APC. The trend was increasing when the AAPC and APC were positive and significantly different from zero. The trend was decreasing when these values were negative and significantly different from zero. When the negative or positive values were not significantly different from zero, the trend was classified as stationary. For both the AAPC and APC analysis, a statistical significance level of less than 0.05 (p < 0.05) and 95% confidence intervals were considered^22,23^.

As the inflection point regression model assumes a linear trend between the points, with the same assumptions as linear regression, except for homoscedasticity and non-autocorrelation, they were incorporated into the Joinpoint software configuration, i.e. a weighted regression model was adjusted^23^. To do this, the first-order autocorrelated option estimated from the data was selected. In this way, the program assumed that the random errors were autocorrelated and estimated the regression coefficients by weighted least squares^22^.

To validate the regression model, tests were carried out to verify three important assumptions: normality, homoscedasticity, and non-autocorrelation of the residuals.

The Kolmogorov-Sminorv and Shapiro-Wilk normality tests were carried out to reveal the non-normal distribution of the residuals, with a p-value < 0.05 in both tests. Non-normality was concluded if p < 0.05, rejecting the null hypothesis. If p > 0.05, normality was not rejected, indicating that the data could be considered normally distributed^24^.

Homoscedasticity, which assumes that the variance of the residuals is constant, was checked using the Breusch-Pagan test, which tests the null hypothesis of homoscedasticity against alternative forms of heteroscedasticity. The null hypothesis was homoscedasticity, where for p-values < 0.05, homoscedasticity is rejected at a significance level of 5%. The assumption that the residuals are not autocorrelated, i.e. that they are independent over time, was assessed using the Durbin-Watson test, which checks for the existence of first-order autocorrelation^25^.

In order to analyze the temporal patterns of the statistical data, we also used the PC calculation, done year by year, considering the year after and the year before, and the PC between the final year and the initial year of the time series, which are widely used in this type of analysis. In other words, the amplitude of the variation over the period 1996 to 2021 was calculated, as well as between each pair of consecutive years in that period. The result was expressed as a percentage using the following formula:

Percentage change, calculation method:


PC=[( value at later time ÷ value at earlier time )−1]×100 (2)

Thus, PC, APC for different line segments, and APC for a line segment were the measures used to analyze the changes and trends of increase, decrease and stability in the indices of the indicators evaluated. The statistical analysis used Microsoft Office Excel application version 2019, Statistics and Data Science (STATA) software, version 17, and Joinpoint Regression software, version 5.10.

### Limitations, Operational Problems, and Ways to Minimize Them

One of the limitations to calculating the FMR is the fact that fetal deaths are recorded from different sources, which can lead to variable quality of information and varying completeness of records in SIM and SINASC. In an information system, completeness refers to the degree to which each analyzed field is filled in, measured by the proportion of filled and unfilled fields^26^.

The completeness of the data is an important quality indicator, since incomplete or unfilled fields can compromise the reliability and accuracy of the analyses carried out on the basis of this information^27^. Non-completeness of data, characterized by incomplete, unfilled or “ignored” records for sociodemographic and obstetric variables and causes of stillbirth, had an impact on the results obtained.

The inclusion or exclusion of these variables in the analysis was carried out according to the following classification of data completeness: excellent (over 95% completeness); good (90 to 95%); fair (80 to 90%); poor (50 to 80%); and very poor (below 50%)^27^. Variables with completeness below 50% were excluded from the evaluations. Some fetal DC data was not recorded, including the race/color variable, which is essential for guiding preventive interventions.

In this case, the “race/color” variable had a high prevalence of incomplete information and was therefore excluded from subsequent analyses. In these analyses, incomplete data ranged from 1.82% for the “place of occurrence” variable to 95.8% for the “race/color” variable. The category “poorly-defined causes” referring to Chapter XVI of ICD-10 exceeded 29% as a record of incomplete information, which compromises fetal mortality statistics^26^. The absence of almost 96% of the records for a critical variable such as race/color is exceptionally high and could have introduced a substantial bias into the statistical analyses.

For the other variables included in this study, the percentage of “ignored” or missing data ranged from 1.82% to 32.95% (maternal education). However, to minimize biases resulting from unfilled records, missing data was imputed. To do this, we used the method of imputation by proportional distribution, adopted by the Brazilian Institute of Geography and Statistics (IBGE), which consisted of grouping cases with ignored or undeclared information, followed by calculating the proportions of the known categories. The cases with unknown information were redistributed proportionally, preserving the original proportions of valid categories^28^.

Cautiously, considering the limitations associated with imputation, sensitivity analyses were carried out to assess the impact of imputation on the main results. The sensitivity analysis to assess the impact of imputation on the results considered whether both samples had similar distributions. To this end, the Kolmogorov-Sminorv and Shapiro-Wilk tests were carried out before and after imputation^28^. No p-values were found below the significance level (p = 0.05), concluding that the distributions did not change after the imputation procedure.

In addition, the sensitivity analysis assessed the impact of imputing missing values on fetal mortality indices and trend patterns. A comparison was made between the index before imputation and the new time series recalculated after the imputation procedure. The information revealed that the new results were higher than the original ones without imputation, throughout the period analyzed. However, they showed a trajectory that followed the previous time series and the same trend pattern.

### Ethical and Legal Aspects

This study complied with the ethical precepts guaranteed in the Declaration of Helsinki and in the Brazilian resolutions No. 466/12 and No. 510/16 of the National Health Council, which deal with research on human beings.

It was not necessary to submit the study project to the Human Research Ethics Committee (CEP), since this is secondary data, without identifying the participants, as provided for in National Health Council (CNS) Resolution No. 466, of December 12, 2012, and CNS Resolution No. 510, of April 7, 2016. The latter stipulates that studies using information in public domain do not need to be submitted to a research CEP.

## RESULTS

### National Trends

Brazil recorded an average of 94 fetal deaths every day, which corresponds to approximately 34,111 cases per year between 1996 and 2021. There were 886,878 stillbirths with a GA of 20 weeks or more in the country, representing an overall fetal mortality rate of 11.4 deaths per 1,000 total births. Of this total, 94% (835,153 cases) were concentrated in the GA ≥ 22 weeks, equivalent to an overall FMR of 10.7/1,000, and 75% (665,287 cases) occurred with GA ≥ 28 weeks, making up an overall FMR of 8.6/1,000.

The average number of stillbirths per year in GA ≥ 20, ≥ 22, and ≥ 28 weeks strata were 34,111, 32,121, and 25,588, with standard deviations of ± 3,958, ± 4,144, and ± 4,633, respectively. The variability around the annual average of stillbirths in the same GA strata was 11.60%, 12.90%, and 18.11%, respectively.

The absolute number of stillbirths gradually decreased, from 40,434, 39,892, and 37,823 cases, respectively, in 1996, to 29,325, 27,022, and 20,186 in 2021, considering GA ≥ 20, ≥ 22, and ≥ 28 weeks. The annual proportion of stillbirths in relation to the total for the period, in the GAs analyzed, showed a downward trend over the years, falling from 4.56%, 4.78%, and 5.69% in 1996 to 3.31%, 3.24%, and 3.03% in 2021, respectively.

The average annual fetal mortality rate for GA ≥ 20 weeks was 11.3 (±0.9 standard deviation). For GA ≥ 22 weeks, this rate was 10.7 (±1.0 SD), while for GA ≥ 28 weeks, it was 8.5 (±1.3 SD). Considering GA ≥ 20 weeks, the probability of a fetus being stillborn per 1,000 total births was 1.06 times higher than for GA ≥ 22 weeks and 1.33 times higher than for GA ≥ 28 weeks. On the other hand, the average annual FMR for GA ≥ 22 weeks was 1.25 times higher than that observed for GA ≥ 28 weeks.

The estimated annual FMRs showed a reduction in the risk of fetal death and an increase in perinatal survival. The Figure shows the time series of the estimated FMR, considering the strata of GA ≥ 20, ≥ 22 and ≥ 28 weeks, in Brazil, between 1996 and 2021. The data indicates a long-term upward trend for FWS, but with distinct fluctuations and periods of increase or decrease in specific segments of the time series, variations which are shown in Table 1.

**Figure:**
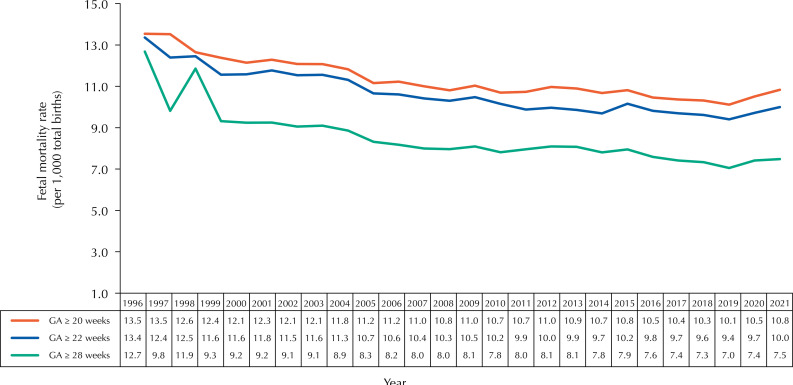
Time series of fetal mortality rates in gestational age strata ≥ 20, ≥ 22, and ≥ 28 weeks. Brazil, 1996–2021.

GA: gestational age.

Source: based on data from the Ministry of Health and Mortality Information System, 2022.

**Table 1:** Analysis of long-term and interrupted trends in the time series of fetal deaths and fetal mortality rates in the gestational age strata ≥ 20, ≥ 22, and ≥ 28 weeks for before and including the covid-19 pandemic. Brazil, 1996–2021.

Variables	Segment	APC	95% CI	AAPC	95% CI	Interpretation
Fetal deaths at gestational age ≥ 20 weeks	1996–1999	0.13	(-1.37 to 2.80)			Increasing
	1999–2008	-2.81^*^	(-3.48 to 2.50)			Increasing
	2008–2014	0.29	(-0.34 to 1.87)			Decreasing
	2014–2021	-1.60^*^	(-2.34 to -1.15)			Increasing
	1996–2019			-1.37^*^	(-1.49 to -1,22)	Decreasing
	1996–2021			-1.39^*^	(-1.52 to -1.25)	Decreasing
Fetal deaths at gestational age ≥ 22 weeks	1996–2003	-1.50^*^	(-1.97 to -0.82)			Decreasing
	2003–2006	-4.29^*^	(-4.90 to -1.84)			Decreasing
	2006–2012	-1.39^*^	(-3.11 to -0.56)			Decreasing
	2012–2015	1.14	(-0.52 to 1.84)			Stationary
	2015–2021	-1.98^*^	(-2.85 to -1.46)			Decreasing
	1996–2019			-1.58^*^	(-1.67 to -1.48)	Decreasing
	1996–2021			-1.61^*^	(-1.73 to -1.51)	Decreasing
Fetal deaths at gestational age ≥ 28 weeks	1996–2008	-3.51^*^	(-3.96 to 3.15)			Decreasing
	2008–2014	0.59	(-0.51 to 3.60)			Stationary
	2014–2021	-2.61^*^	(-4.55 to -1.76)			Decreasing
	1996–2019			-2.26^*^	(-2.42 to -2.10)	Decreasing
	1999–2021			-2.29^*^	(-2.50 to -2.12)	Decreasing
Fetal mortality rate at gestational age ≥ 20 weeks	1996–2007	-1.77^*^	(-3.10 to -1.27)			Decreasing
	2007–2021	-0.4	(-0.74 to 0.46)			Decreasing
	1996–2019			-1.06^*^	(-1.23 to -0.89)	Decreasing
	1996–2021			-1.00^*^	(-1.19 to -0.81)	Decreasing
Fetal mortality rate at gestational age ≥ 22 weeks	1996–1999	-3.99^*^	(-6.30 to -2.75)			Decreasing
	1999–2003	-0.28	(-1.12 to 1.03)			Stationary
	2003–2006	-3.19^*^	(-3.85 to -1.95)			Decreasing
	2006–2019	-0.75^*^	(-1.03 to -0.56)			Decreasing
	2019–2021	2.23	(-0.01 to 3.66)			Stationary
	1996–2019			-1.42^*^	(-1.53 to -1.28)	Decreasing
	1996–2021			-1.13^*^	(-1.29 to -1.01)	Decreasing
Fetal mortality rate at gestational age ≥ 28 weeks	1996–2000	-5.44^*^	(-10.11 to -2.51)			Decreasing
	2000–2007	-1.9	(-4.45 to 1.29)			Stationary
	2007–2021	-0.85	(-5.32 to 1.94)			Stationary
	1996–2019			-1.98^*^	(-2.23 to -1.61)	Decreasing
	1996–2021			-1.89^*^	(-2.27 to -1.53)	Decreasing

95%CI: 95% confidence interval.

Source: based on data from the Ministry of Health and Mortality Information System, 2022.

* Indicates that the annual percentage change (APC) and the average annual percentage change (AAPC) are significantly different from zero at the alpha = 0.05 level. The interpretation of an interrupted trend is given by the APC, and of a long-term trend is given by the AAPC.

Table 1 shows the analysis of long-term trends and interrupted trends in the time series of stillbirths and FMR in the GA ≥ 20, ≥ 22 and ≥ 28 weeks strata in Brazil between 1996 and 2021. These trends are analyzed by calculating the APC and AAPC.

The absolute number of stillbirths at GA ≥ 20 weeks fell by 28% (from 40,434 in 1996 to 29,325 in 2021), with an AAPC of -1.39. The reduction was more pronounced for GA ≥ 22 weeks, down 32% (AAPC -1.62; from 39,892 to 27,022), and for GA ≥ 28 weeks, down 47% (AAPC -2.29; from 37,823 to 20,186). In the GA ≥ 20 weeks, the 1996 to 2008 and 2014 to 2021 segments stood out, with an upward trend. For GA ≥ 22 weeks, there was a period of stability between 2012 and 2015, followed by a decrease from 2015 to 2021. For GA ≥ 28 weeks, there was stability from 2008 to 2014, with a resumption of the reduction from 2014 to 2021.

During the covid-19 pandemic, the number of stillbirths showed specific variations by GI strata analyzed. In GA ≥ 20 weeks, there was a decrease of 0.38% in 2020, followed by an increase of 1.15% in 2021, resulting in an overall increase of 0.76% in the biennium. On the other hand, for GA ≥ 22 weeks, there was a 1% reduction in 2020 and a 0.9% increase in 2021, leading to a 0.09% reduction in the biennium. As for GA ≥ 28 weeks, stillbirths increased by 0.75% in 2020 and decreased by 0.94% in 2021, culminating in a reduction of 0.19% in the biennium.

The FMR in the GI ≥ 20, ≥ 22, and ≥ 28 weeks strata showed a long-term downward trend over the period analyzed. The AAPCs were -1.00, -1.13, and -1.89, respectively, and were statistically significant. Notably, the AAPC of the FMR time series at GA ≥ 20 weeks was 12% lower than at GA ≥ 22 weeks and 47% lower than at GA ≥ 28 weeks. FRM decreased by 20%, 25%, and 41%, respectively, in the GA strata analyzed between 1996 and 2021.

Before the covid-19 pandemic (1996–2019), the negative AAPC of the FMR time series was 1.06, 1.26, and 1.05 times higher, respectively, in the GA strata analyzed, compared to the negative AAPC seen in the period including the pandemic (1996–2021) in the same strata. However, the long-term downward trend in fetal mortality was not interrupted. In the analysis of interrupted trends, specific periods of increase, decrease or stabilization were observed in the FMR time series in the GA strata analyzed.

### Sociodemographic and Obstetric Determinants

The data reveal disparities and inequalities in fetal mortality influenced by demographic and obstetric characteristics in Brazil between 1996 and 2021. Table 2 shows the overall fetal mortality rates and the long-term trend analysis in the time series of annual FMR in the strata of GA ≥ 22 and ≥ 28 weeks by sociodemographic characteristics, for the periods before and including the covid-19 pandemic in Brazil, between 1996 and 2021. Despite the reductions observed, inequalities in fetal mortality associated with the sex of the fetal death, maternal age, and maternal schooling persisted.

**Table 2:** Overall fetal mortality rate and analysis of long-term trends in the time series of fetal mortality rates in the gestational age strata ≥ 22 and ≥ 28 weeks by sociodemographic characteristics for before and including the covid-19 pandemic. Brazil, 1996–2021.

Variables	Fetal mortality							
	Gestational age ≥ 22 weeks				Gestational age ≥ 28 weeks			
	1996–2019^**^		1996–2021^***^		1996–2019^**^		1996–2021^***^	
	FMR^****^	AAPC^*^	FMR^****^	AAPC^*^	FMR^****^	AAPC^*^	FMR^****^	AAPC^*^
Sex of death								
Male	11.3	-1.19^*^	11.3	-1.12^*^	9.1	-2.07^*^	9	-2.00^*^
Female	10.1	-1.01^*^	10.1	-0.93^*^	8.2	-1.61^*^	8.1	-1.53^*^
Maternal age (years)								
10–14	13.6	-0.36^*^	13.7	-0.22^*^	9.2	-1.43^*^	9.2	-1.26^*^
15–19 years	10.2	-0.55^*^	10.3	-0.40^*^	7.8	-1.41^*^	7.8	-1.26^*^
20–24 years	9.6	-0.64^*^	9.5	-0.59^*^	7.6	-1.58^*^	7.6	-1.56^*^
25–29 years	9.7	-1.26^*^	9.6	-1.39^*^	7.9	-1.88^*^	7.8	-1.80^*^
30–34 years	11	-2.17^*^	10.8	-1.72^*^	9	-2.85^*^	8.8	-2.46^*^
35–39 years	14.8	-2.86^*^	14.4	-2.51^*^	12.3	-3.36^*^	11.9	-3.33^*^
40–44 years	23	-2.65^*^	22.3	-2.77^*^	19.7	-3.07^*^	18.9	-3.07^*^
45–49 years	34.1	-1.64^*^	33.1	-1.77^*^	29.8	-2.15^*^	28.8	-2.27^*^
Maternal schooling (in years)								
None	31.7	9.45^*^	32.4	9.21^*^	24.6	8.30^*^	25.1	8.03^*^
1–3 years	13.4	7.35^*^	13.6	7.16^*^	10.2	6.62^*^	10.4	6.40^*^
4–7 years	12.6	-1.94^*^	12.7	-1.54^*^	9.2	-2.96^*^	9.4	-2.48^*^
8–11 years	9.2	-2.89^*^	9.1	-2.65^*^	8.4	-4.17^*^	8.2	-3.93^*^
≥ 12 years	7.2	-0.77^*^	7.1	-0.69^*^	4.9	1.60^*^	4.8	-1.07^*^

^*^Indicates that the average annual percentage change (AAPC) is significantly different from zero at the alpha = 0.05 level, if positive, an increasing trend, if negative, a decreasing trend. When the positive or negative AAPC is not significantly different from zero, stability. ^**^Period prior to the covid-19 pandemic. ^***^Period that included the covid-19 pandemic. ^****^Global fetal mortality rate per 1,000 total births (live births + stillbirths) for the total periods: 1996–2019 and 1996–2021.

Source: based on data from the Ministry of Health and Mortality Information System, 2022.

The pattern of global fetal mortality rates for GA ≥ 22 and GA ≥ 28 weeks is similar, with lower global FMR at GA ≥ 28 weeks, in the total period from 1996 to 2021. The estimated overall risk of a fetus being born without any sign of life per 1,000 total births (live births + stillbirths), in the period analyzed, was higher for males than for females, both in the period before and in the period including the covid-19 pandemic.

At the extremes of maternal age, i.e. pregnant women of advanced age, especially between 45 and 49 years, and adolescents, especially between 10 and 14 years, the estimated risk of fetal mortality was higher. Overall FMRs were 3.5 times higher in the older maternal age group (45–49 years), compared to the lower rates observed in the 20–24 age group, when considering GA ≥ 22 weeks. In the case of GA ≥ 28 weeks, the overall FMR in the oldest maternal age group (45–49 years) was 3.8 times higher than the minimum rates recorded in the 20–24 age group.

In addition, pregnant women with no schooling had a higher risk of fetal mortality compared to those with some level of education, and there was a trend towards progressively lower FMRs as the level of education increased. Overall FMRs were particularly high among women with no formal schooling, being 4.5 times higher at GA ≥ 22 weeks and 5.2 times higher at GA ≥ 28 weeks, when compared to the minimum FMRs recorded for women with 12 years or more of schooling.

Regarding the trend pattern, although FMRs were higher in males, there was a more marked reduction in these rates compared to females. There was a significant long-term downward trend in FMR for all age groups of mothers and levels of maternal schooling, except for women with no schooling and one to three years of schooling, who showed an upward trend. The greatest reductions in overall FMR in relation to maternal age occurred in the 25–44 age groups.

When comparing the periods before (1996–2019) and including the covid-19 pandemic, it was observed that the overall FMR remained practically unchanged for both sexes of fetal death, maternal age groups, and maternal education levels. However, the negative AAPC, which indicates a downward trend in FMR, was lower in the period including the pandemic for most of the groups analyzed, suggesting a slowdown in the decline of fetal mortality. This pattern of deceleration in the decline was similar for the GA strata analyzed.

The deceleration in the long-term downward trend in the overall FMR for the period can be explained by the significant increases in these rates observed in 2020, especially in the most vulnerable socio-demographic strata. These increases were followed by smaller increases in 2021, accompanied by some occasional reductions.

Table 3 shows the overall fetal mortality rates and the analysis of long-term trends in the annual FMR time series, considering the GA strata (≥ 22 and ≥ 28 weeks), by obstetric characteristics, for the periods before and including the covid-19 pandemic in Brazil, between 1996 and 2021. There was inequality in the risk of fetal mortality associated with the place of occurrence of fetal death, the duration and type of pregnancy, the type of delivery and birth weight in the country.

**Table 3:** Overall fetal mortality rate and analysis of long-term trends in the time series of fetal mortality rates in the gestational age strata ≥ 22 and ≥ 28 weeks according to obstetric characteristics for before and including the covid-19 pandemic. Brazil, 1996–2021.

Variables	Fetal mortality							
	Gestational age ≥ 22 weeks				Gestational age ≥ 28 weeks			
	1996–2019^**^		1996–2021^***^		1996–2019^**^		1996–2021^***^	
	FMR^****^	AAPC^*^	FMR^****^	AAPC^*^	FMR^****^	AAPC^*^	FMR^****^	AAPC^*^
Place of occurrence								
Hospital	10.3	-1.12^*^	10.3	-1.05^*^	8.3	-2.10^*^	8.2	-2.03^*^
Other health establishment	11.9	1.36^*^	12.3	1.83^*^	10	0.57^*^	10.3	1.05
Home	34.8	0.42^*^	35.4	0.51^*^	29	-0.3	29.5	-0.19^*^
Other	119.5	-3.57^*^	109.2	-4.38^*^	105	-3.97^*^	95.6	-4.76^*^
Length of pregnancy (weeks)								
22–27	381.2	-0.01	378	0.08	-	-	-	-
28–31	207.5	-4.07^*^	203.2	-3.77^*^	218	-3.97^*^	213.1	-3.69^*^
32–36	41.5	-3.37^*^	40.2	-3.11^*^	44	-4.30^*^	42.6	-3.98^*^
37–41	3.2	-0.86^*^	3.2	-0.94^*^	3.4	-1.42^*^	3.4	-1.37^*^
≥ 42	7.5	-6.30^*^	7.1	-5.96^*^	8	-6.47^*^	7.6	-6.11^*^
Type of pregnancy								
Unique	10.4	-1.10^*^	10.3	-1.03^*^	8.4	-1.90^*^	8.3	-1.81^*^
Double	28.5	-1.67^*^	28.1	-1.61^*^	21	-3.09^*^	20.5	-3.06^*^
Triple and more	41.6	-2.86^*^	41	-2.57^*^	31.1	-4.59^*^	30.5	-4.26^*^
Type of delivery								
Vaginal	14.4	-0.29^*^	14.4	0.02^*^	10.8	-1.43^*^	10.8	-1.03^*^
Cesarean section	6.7	-2.08^*^	6.6	-1.70^*^	6.2	-2.42^*^	6.1	-2.00^*^
Birth weight (g)								
< 500	220.4	-0.12	230.5	0.25	92.3	-4.01^*^	91.2	-3.75^*^
500–999	354.7	-0.53^*^	352.7	-0.49^*^	170.7	-2.47^*^	168.7	-2.30^*^
1,000–1,499	191	-1.24^*^	189.3	-0.92^*^	160.2	-1.53^*^	159.1	-1.34^*^
1,500–2,499	39.4	-1.25^*^	39.1	-1.21^*^	39.1	-1.38^*^	38.8	-1.32^*^
2,500–2,999	5.6	-1.43^*^	5.6	-1.37^*^	5.7	-1.57^*^	5.7	-1.51^*^
3,000–3,999	2.5	-1.99^*^	2.5	-1.99^*^	2.6	-2.17^*^	2.5	-2.17^*^
≥ 4,000	6.4	-2.09^*^	6.3	-2.14^*^	6.5	-2.23^*^	6.4	-2.30^*^

FMR: fetal mortality rate; g: grams.

Source: based on data from the Ministry of Health and Mortality Information System, 2022.

^*^Indicates that the average annual percentage change (AAPC) is significantly different from zero at the alpha = 0.05 level, if positive, an increasing trend, if negative, a decreasing trend. When the positive or negative AAPC is not significantly different from zero, stability. ^**^Period prior to the covid-19 pandemic. ^***^Period that included the covid-19 pandemic. ^****^Global fetal mortality rate per 1,000 total births (live births + stillbirths) for the total periods: 1996–2019 and 1996–2021.

Similar to what was observed for the sociodemographic characteristics of fetal deaths (Table 2), by obstetric variables, the highest overall fetal mortality rates for the period analyzed, considering both GA strata analyzed in Table 3, occurred for GA ≥ 22 weeks, when compared to GA ≥ 28 weeks. For pregnancies ≥ 20 and < 22 weeks, although not included in Table 3, overall FMRs were even higher compared to GA ≥ 22 and ≥ 28 weeks.

In the groups of obstetric variables, the highest overall FMR for the period 1996 to 2021 occurred in home births and other locations, such as indigenous villages and public roads. In addition, the overall FMR was higher in pregnancies lasting less than 37 weeks and was even higher in those lasting less than 31 weeks, as well as in twin pregnancies and triplets or more. Higher overall FMRs were also observed in vaginal and low birth weight stillbirths (< 2,500 g), with a higher concentration in the weight range between 500 and 999 g.

In relation to the place where the fetal death occurred, the highest overall FMR in other places, such as public roads and indigenous villages, was 11.6 times higher than the lowest overall FMR observed in hospitals. The highest overall FMR in pregnancies between 22 and 27 weeks was 119 times higher than the lowest observed in pregnancies between 37 and 41 weeks. In twin and trigeminal pregnancies, the overall FMR was approximately four times higher compared to single pregnancies. In addition, overall FMR was twice as high in vaginal deliveries compared to cesarean deliveries. As for birth weight, the highest overall FMR in the 500 to 999 g range was 142 times higher than in the 3,000 to 3,999 g range.

Significant long-term downward trends were observed in most categories of the groups of obstetric characteristics analyzed. Annual FMR showed the greatest reductions in other locations and hospitals, in pregnancies of 42 weeks or more, and between 28 and 31 weeks, in multiple twin pregnancies, in cesarean deliveries and in birth weights of 4,000 g or more. Specifically, in the stratum of GA ≥ 28 weeks, the annual FMR decreased dramatically for birth weight < 500 g. The exceptions to the increase in annual FMR were for other health facilities and home births (for GA ≥ 22 weeks), and a stationary trend was observed for birth weight < 500 g.

When comparing the periods before (1996–2019) and including the pandemic (1996–2021), it was observed that both the overall FMR and the trends in the annual FMR time series were quite similar for most of the categories analyzed. However, the covid-19 pandemic had a significant impact, causing a slowdown in long-term downward trends and an acceleration in upward trends, as observed in another health facility.

### Perinatal Factors Responsible for Fetal Complications

The fetal complications responsible for causing most fetal deaths were triggered by perinatal factors and congenital malformations, the impact of which could be mitigated by improvements in prenatal care and during childbirth, both in primary care and in hospital care. Table 4 shows the total number of stillbirths, the corresponding proportion, and the long-term trend analysis in the annual time series of stillbirths in the strata of GA ≥ 22 and ≥ 28 weeks by chapter and list of causes of death of the ICD-10, in Brazil, for the period 1996 to 2021.

**Table 4:** Total number of fetal deaths, proportion and trend analysis in the time series of fetal deaths in the gestational age strata ≥ 22 and ≥ 28 weeks before and including the covid-19 pandemic by chapter and list of causes of death of ICD-10. Brazil, 1996–2021.

Chapter and list of causes of death ICD-10	Fetal mortality									
	Gestational age ≥ 22 weeks					Gestational age ≥ 28 weeks				
	1996–2019^**^		1996–2021^***^			1996–2019^**^		1996–2021^***^		
	n^****^	AAPC^*^	n^****^	(ρ)^*****^	AAPC^*^	n^****^	AAPC^*^	n^****^	(ρ)^*****^	AAPC^*^
Some infectious and parasitic diseases	4,394	11.46^*^	5,220	-0.65	10.56^*^	3,397	13.41^*^	4,064	-0.65	12.43^*^
Neoplasms	2	-	2	-0.0003	-	2	-	2	-0.0003	-
Some conditions originating in the perinatal period	699,815	0.23	749,265	-94	0.13	554,368	-0.16	591,847	-94.05	-0.28
Fetus and newborn affected by maternal factors and complications of pregnancy, labor, and delivery	275,748	1.77^*^	299,793	-37.61	1.70^*^	216,894	1.43^*^	234,839	-37.32	1.36^*^
Disorders related to the length of pregnancy and fetal growth	10,381	-6.03^*^	11,201	-1.41	-3.65^*^	7,022	-6.17^*^	7,527	-1.2	-5.80^*^
Birth trauma	563	2.35	583	-0.07	1.8	490	2.29	509	-0.08	1.77
Intrauterine hypoxia and birth asphyxia	187,255	-0.83^*^	196,976	-24.71	-1.72^*^	148,838	-1.37^*^	156,230	-24.83	-1.90^*^
Newborn respiratory distress	119	-10.42^*^	119	-0.01	-10.42^*^	92	-9.57^*^	92	-0.01	-9.57^*^
Congenital pneumonia	41	-15.05^*^	41	-0.01	-14.02^*^	40	-15.13^*^	40	-0.01	-14.10^*^
Other respiratory conditions of the newborn	1,103	-13.29^*^	1,124	-0.14	-12.44^*^	979	-13.47^*^	996	-0.16	-12.63^*^
Bacterial septicemia of the newborn	42	-7.02^*^	42	-0.01	-7.02^*^	36	-6.85^*^	36	-0.01	-6.85^*^
Hemorrhagic and hematological disorders of the fetus	2,911	-1.01^*^	3,184	-0.4	-0.24	2,335	-1.96^*^	2,514	-0.4	-1.96^*^
Other perinatal conditions	221,652	-0.67^*^	236,202	-29.63	-0.41	177,642	-0.85^*^	189,064	-30.05	-0.5
Congenital malformations, deformities and anomalies	38,067	2.48^*^	41,593	-5.22	2.40^*^	30,007	1.59^*^	32,424	-5.15	1.50^*^
Congenital hydrocephalus and spina bifida	2,090	-2.26^*^	2,172	-0.27	-3.34^*^	1,700	-3.52^*^	1,755	-0.28	-4.08^*^
Other congenital malformations of the nervous system	9,730	0.6	10,351	-1.3	0.45	7,938	-0.24	8,338	-1.33	-0.53
Congenital heart defects	3,288	4.11^*^	3,695	-0.46	4.11^*^	2,769	3.41^*^	3,084	-0.49	3.41
Other congenital malformations of the circulatory system	362	8.87^*^	391	-0.05	4.85^*^	287	5.78^*^	308	-0.05	1.69
Down’s syndrome and other chromosomal anomalies	2,500	10.49^*^	2,985	-0.37	10.26^*^	1,900	9.92^*^	2,243	-0.36	9.65^*^
Other congenital malformations	20,097	2.45^*^	21,999	-2.76	2.37^*^	15,413	1.45^*^	16,696	-2.65	1.21^*^
Symptoms, signs, and abnormal clinical examination findings	972	-22.85^*^	972	-0.12	-21.25^*^	917	-22.68^*^	917	-0.15	-21.09^*^
Sudden infant death syndrome	10	-2.25	10	-0.001	-2.05	9	-2.33	9	-0.001	-2.11
Other symptoms, signs, and abnormal test findings	962	-22.81^*^	962	-0.12	-21.17^*^	908	-22.62^*^	908	-0.14	-21.00^*^
All other diseases	1	-	1	-0.0001	-	1	-	1	-0.0002	-
External causes of morbidity and mortality	8	-9.11^*^	8	-0.001	-8.45^*^	8	-9.11^*^	8	-0.001	-8.45^*^

^*^Indicates that the average annual percentage change (AAPC) is significantly different from zero at the alpha = 0.05 level, if positive, an increasing trend, if negative, a decreasing trend. When the positive or negative AAPC is not significantly different from zero, stability. ^**^Period prior to the covid-19 pandemic. ^***^Period that included the covid-19 pandemic. ^****^Total population of fetal deaths by chapter and list of causes of death of ICD-10 in the strata of gestational age ≥ 22 and ≥ 28 weeks. ^*****^Proportion.

Source: based on data from the Ministry of Health and Mortality Information System, 2022.

The main causes of fetal deaths in the GA strata analyzed were conditions originating in the perinatal period (94%) - Chapter XVI of ICD-10, with “Fetus and newborn affected by maternal factors and by complications of pregnancy, labor, and delivery” (38%) and “Intrauterine hypoxia and birth asphyxia” (25%) standing out. Congenital malformations, deformities and chromosomal anomalies (Chapter XVII of ICD-10) also represented a relevant cause (5%), with “Other congenital malformations of the nervous system” (1.3%) and “Down’s syndrome and other chromosomal anomalies” (0.37%) standing out.

There was a long-term upward trend in fetal deaths related to the groupings of causes by ICD-10 chapters, with the exception of Chapter XVIII (“Symptoms, signs, and abnormal clinical and laboratory findings, not elsewhere classified”), which showed a downward trend, and Chapter XVI, which remained stable. During the period of the covid-19 pandemic (2020–2021), there was a slowdown in the upward and downward trends in the groupings by chapter of causes of fetal deaths.

The long-term upward trend in the number of fetal deaths, broken down by the list of causes of death, was specifically concentrated in perinatal conditions, particularly in the group “fetus and newborn affected by maternal factors and complications of pregnancy, of labor and delivery” (AAPC of 1.70 at GA ≥ 22 weeks and 1.36 at GA ≥ 28 weeks), and congenital malformations, with a greater increase in fetal deaths due to “Down’s syndrome and other chromosomal anomalies” (AAPC of 10.26 at GA ≥ 22 weeks and 9.65 at GA ≥ 28 weeks). This upward and downward trend was attenuated during the covid-19 pandemic.

A significant proportion of fetal deaths (67%) in Brazil between 1996 and 2021 were potentially preventable, with 39% being reducible through adequate care for pregnant women during pregnancy and childbirth in Primary Health Care, and 28% preventable with qualified hospital care during childbirth. Immunoprevention actions could have reduced 0.65% of fetal deaths classified in Primary Health Care. These data highlight the importance of investments and improvements in prenatal care, childbirth, and preventive measures to reduce fetal mortality in the country during the period analyzed.

### Regional Differences

The data analyzed reveals regional differences in the distribution of stillbirths and stillbirths in Brazil between 1996 and 2021. The Southeast and Northeast regions concentrated the highest absolute numbers of stillbirths in the country, considering GA ≥ 20 and ≥ 22 weeks, respectively. The Southeast had an annual average of 13,483 (± 3,014 standard deviation) and 12,581 (± 2,905 SD) stillbirths, while the Northeast had an annual average of 10,996 (± 924 SD) and 10,416 (± 995 SD) stillbirths.

Although with lower absolute numbers, the North and Northeast regions had the highest relative gross average rates of stillbirths per 1,000 total births in the GA strata analyzed. The North had an annual average of 11.7 (± 11.7 SD) and 11.0 (± 0.9 SD) stillbirths per 1,000 total births, while the Northeast recorded an annual average of 12.6 (± 0.6 SD) and 12.0 (± 0.6 SD) stillbirths per 1,000 total births, for GA ≥ 20 and ≥ 22 weeks, respectively.

The Central-West region had the lowest absolute number of stillbirths, with an annual average of 2,331 (± 156 standard deviation) and 2,207 (± 180 SD), considering GA ≥ 20 and ≥ 22 weeks, respectively. However, the South had the lowest crude fetal mortality rate, with an annual average of 9.1 (± 1.1 SD) and 8.7 (± 1.3 SD) deaths per 1,000 total births, respectively, for the same GA strata analyzed.

The North and South regions had a median absolute number of stillbirths, with 3,562 (± 132 standard deviation) and 3,343 (± 159 SD), and 3,738 (± 768 SD), and 3,573 (± 811 SD), respectively, considering GA ≥ 20 and ≥ 22 weeks. However, it was the Southeast and Central-West regions that had the highest median crude FMR, with an annual average of 11.3 (± 1.9 SD) and 10.6 (± 1.9 SD) stillbirths per 1,000 total births in the Southeast region, and 9.9 (± 0.6 SD) and 9.4 (± 0.7 SD) stillbirths per 1,000 total births in the Central-West region, in the same GA strata analyzed. The higher absolute number of stillbirths in the Southeast can be attributed to the larger population (42.28%) and higher number of births (39.43%) in this region.

The standardized average annual FMR, considering GA ≥ 22 weeks, adjusted by Brazil’s national parameters, of the North (10.4 deaths per 1,000 total births; standard deviation ± 0.9), Northeast (10.6/1,000; SD ±1.0), and Southeast (10.5 ± 1.9 SD) regions were similar to and below the national average FMR (10.7/1,000; SD ±1.0) between 1996 and 2021. However, the standardized annual average FMR in the Northeast region was higher than in the Southeast, North, South (8.6 ± 1.2 SD) and Central-West (9.7 ± 0.9 SD) during the period analyzed. From 1996 to 2000 and in 2002, the standardized MFR in the Southeast was higher than the national MFR.

All regions showed a general downward trend in the number of fetal deaths over the period analyzed (1996–2021), considering GA ≥ 20 and ≥ 22 weeks, with some occasional fluctuations. The Southeast region recorded the sharpest drop, with a reduction of 48% and 51%, respectively.

The North and Northeast regions also showed significant reductions in the number of fetal deaths, although with lower absolute values. The North showed a reduction of 12% and 4%, while the Northeast showed a drop of 3% and 5%, considering GA ≥ 20 and ≥ 22 weeks, respectively. The South had the second largest reduction, with 41% and 46%, and the Central-West had a median reduction of 3% and 5.6%, respectively.

Table 5 shows the analysis of the long-term trend and interrupted trend in the time series of annual crude fetal mortality rates for the years prior to and including the years of the covid-19 pandemic, according to the Major Regions in Brazil, between 1996 and 2021. Most of the country’s regions showed a long-term downward trend in crude FMR over the period analyzed. However, the Central-West region remained stationary during this period, showing no significant variations in gross FMR.

**Table 5:** Long-term trend and interrupted trend analysis in the time series of crude fetal mortality rates before and including the covid-19 pandemic according to the Greater Region. Brazil, 1996–2021.

Variables	Segment	APC	95%CI	AAPC	95%CI	Interpretation
North	1996–2000	-3.45^*^	(-7.31 to -1.45)			Decreasing
	2000–2014	-1.03	(-1.50 to 0.06)			Stationary
	2014–2021	1.23^*^	(0.12 to 4.00)			Increasing
	1996–2019			-0.97^*^	(-1.24 to -0.70)	Decreasing
	1996–2021			-0.79^*^	(-1.06 to -0.51)	Decreasing
North East	1996–1999	-3.55^*^	(-8.14 to -1.24)			Decreasing
	1999–2002	6.28^*^	(3.37 to 7.78)			Increasing
	2002–2021	-0.71^*^	(-0.91 to -0.57)			Decreasing
	1996–2019			-0.21	(-0.39 to 0.03)	Stationary
	1996–2021			-0.25^*^	(-0.42 to -0.03)	Decreasing
South East	1996–1998	-8.83^*^	(-10.84 to -3.92)			Decreasing
	1998–2008	-3.44^*^	(-4.00 to -0.52)			Decreasing
	2008–2021	-0.2	(-0.62 to 0.42)			Stationary
	1996–2019			-2.39^*^	(-2.56 to -2.04)	Decreasing
	1996–2021			-2.22^*^	(-2.39 to -1.89)	Decreasing
South	1996–1999	1.82	(-0.27 to 5.50)			Stationary
	1999–2008	-3.06^*^	(-4.12 to -2.62)			Decreasing
	2008–2021	-1.14^*^	(-1.41 to -0.80)			Decreasing
	1996–2019			-1.52^*^	(-1.68 to -1.32)	Decreasing
	1996–2021			-1.49^*^	(-1.64 to -1.30)	Decreasing
Central-West	1996–2001	1.60^*^	(0.22 to 4.88)			Increasing
	2001–2005	-3.84^*^	(-5.93 to -1.67)			Decreasing
	2005–2019	-0.46	(-1.23 to 0.13)			Stationary
	2019–2021	6.03^*^	(0.07 to 8.96)			Increasing
	1996–2019			-0.61^*^	(-0.79 to -0.23)	Decreasing
	1996–2021			-0.10	(-0.47 to 0.20)	Stationary

*Indicates that the annual percentage change (APC) and average annual percentage change (AAPC) is significantly different from zero at the alpha = 0.05 level. The interpretation of an interrupted trend is given by APC, and of a long-term trend is given by AAC.

95%CI: 95% confidence interval.

Source: based on data from the Ministry of Health and Mortality Information System, 2022.

The smallest reduction in crude FMR, considering GA ≥ 22 weeks, occurred in the Northeast (2%), with an AAPC of -0.25, indicating a long-term downward trend, although it remained stationary between 1996 and 2019. The North region then showed a downward trend, with a 19% reduction in gross FMR, with an AAPC of -0.42; and the Central-West saw a 1% reduction, with an AAPC of -0.79. The regions with the biggest reductions in gross FMR were the Southeast (41%), with an AAPC of -2.04, and the South (29%), with an AAPC of -1.14.

When considering standardized FMRs for GA ≥ 22 weeks, there were more significant reductions than those observed in crude FMRs. In the North, there was a reduction of 23% (AAPC -1.08; 95%CI -1.25 to -0.85), in the Northeast, a reduction of 25% (AAPC -1.23; 95%CI -1.41 to -0.99), and in the Central-West, a reduction of 6.7% (AAPC -0.34; 95%CI -0.76 to -0.05), all statistically significant. On the other hand, the Southeast maintained a similar reduction (41%), while the South showed a reduction of 26% (AAPC -1.38; 95%CI -1.54 to -1.13), less than the reduction seen in gross FMR.

Over the period analyzed, there were intervals in which the FMR did not change significantly in the major regions. In other words, time segments of stabilization of these rates were identified. In the Southeast, the FMR stabilized in the recent period, from 2008 to 2021. In the South, the FMR showed stability between 1996 and 1999. The North region showed stability in the FMR between 2000 and 2014, while the Central-West showed stable rates between 2005 and 2019.

The Southeast and South maintained similar downward trends in FMR before and including the covid-19 pandemic, with a slowdown in the reduction in the pandemic years. On the other hand, the other regions behaved differently. The North showed a slowdown in the reduction of the FMR, while the Northeast went from a stationary trend to a downward trend. The Central-West, on the other hand, reverted from a downward to a stationary trend when data from the pandemic period was included. These data suggest that covid-19 has had different impacts on regional trends in fetal mortality in Brazil.

## DISCUSSION

### National Trends

Brazil recorded substantial progress in fetal survival between 1996 and 2021, however, the results show that fetal mortality is a serious public health problem in the country, with rates considered worrying and information gaps^29,30^. The average annual fetal mortality rates were 11.3, 10.7 and 8.5 stillbirths per 1,000 total births, for GA ≥ 20, ≥ 22 and ≥ 28 weeks, respectively. These rates indicate a high risk of fetal death in the country, higher than that observed in Latin American, Caribbean, and developed countries^9^ and developed countries, calling for interventions and measures to reduce avoidable fetal deaths^12,31^.

This progress was accompanied by a significant drop in fetal deaths with a GA of 20 weeks or more. There was also a lower annual reduction in fetal mortality than in infant mortality. In addition, the long-term downward trend in fetal mortality was impacted by the covid-19 pandemic in 2020 and 2021^32,33^ with a slowdown in the rate of reduction of FMRs, but it has not been interrupted. Before the pandemic (1996–2019), the reduction in FMR was greater than that seen in the period including the pandemic years (1996–2021).

In recent years, from 1996 to 2021, Brazil has recorded a significant number of stillbirths with a GA ≥ 20 weeks, totaling 886,878 cases, equivalent to approximately 70 stillbirths per day. Of this amount, 835,153 (94%) occurred at GA ≥ 22 weeks, and 665,287 (75%) at GA ≥ 28 weeks, considered viable fetuses^34^. Each year, approximately 25,000 stillbirths were recorded at GA ≥ 20 weeks, 32,000 at GA ≥ 22 weeks, or 34,000 at GA ≥ 28 weeks. These data indicate the importance of the need for interventions to reduce stillbirths.

In Latin America and the Caribbean, between 2000 and 2021, the share of Brazilian stillbirths at GA ≥ 28 weeks increased by 17.2%: from 23% in 2000 to 27% in 2021, showing a worsening of the indicator in relation to the regional average and a need for greater attention from the country. In the global scenario, Brazil increased its share of this mortality by 4%, from 1.03% in 2000 to 1.07 in 2021, at GA ≥ 28 weeks, a worrying picture of fetal mortality^9^.

When comparing the FMR in Brazil, considering GA ≥ 28 weeks, which were 12.7 deaths per 1,000 total births in 2000 and 7.5 in 2021, with those of Latin America and the Caribbean, 11.2/1,000 in 2000 and 7.7 in 2021, it was observed that the country had a lower rate in 2021. At the global level, whose rates at GA ≥ 28 weeks were 21.3/1,000 in 2000 and 13.9 in 2021, the country showed lower rates in the period analyzed^9^.

The high percentage of 1.14% of fetal losses with GA ≥ 20 weeks in relation to total births is a worrying indicator that could be mitigated by 67% with effective interventions^35,36^ and adequate public policies, as it reflects failures in maternal and child health systems^35,37^. More worrying is the occurrence of fetal deaths in viable fetuses with GA ≥ 22 weeks, which account for approximately 57% of perinatal deaths from GA ≥ 22 weeks and age 0-6 days. For perinatal deaths from GA ≥ 28 weeks, 51% were fetal deaths, in even more viable fetuses^9^.

The significant presence of fetal deaths in viable fetuses, coupled with possible underreporting, indicates the need for interventions at different stages of the gestational cycle to increase the chances of fetal survival. Monitoring early fetal deaths, especially preventable ones, which require greater attention and investment, is fundamental to the process of preventing and controlling fetal mortality, in order to reduce it^38^.

The progress made in reducing FMR in Brazil over the period analyzed is an important indicator of improvements in maternal and child health, representing advances in fetal and perinatal survival in the country^39^. These rates showed a long-term downward trend, with annual reduction rates of 1.0%, 1.1%, and 1.89% for GA ≥ 20, ≥ 22, and ≥ 28 weeks, respectively. However, it was found that fetal survival rates vary according to GA and determinants.^42^.

Considering GA ≥ 28 weeks, the annual rate of reduction in FMR in Brazil was 0.85% (95%CI -1.0 to -0.69) between 1996 and 2021. This annual rate of reduction in the country represented only 48% of the rate observed in Latin America and the Caribbean, which was 1.78% per year. Compared to the global level, where progress in reducing fetal mortality over the last two decades has been 35% and the global annual rate of reduction has been 2.0%, the annual rate of reduction in FMR in the country has been less than 5%^9^.

These advances in reducing FMR can be attributed to improvements in prenatal care, childbirth care and the general living conditions of the population^43^. Improvements in medical technology have led to greater fetal viability and, consequently, higher fetal survival rates, which has led several international organizations to recommend registering fetal deaths at 20 weeks or more of gestation; however, in order to make comparisons within countries, it has been standardized to define stillbirths at GA ≥ 22 weeks of gestation and/or 500 g or 25 centimeters for the count^44,45^.

In Brazil, the criterion for classifying fetal death is 22 weeks or more of gestation and/or 500 g or 25 cm, a parameter recommended by the WHO adopted in the ICD-10^13^, and is also used in the country to define compulsory notification and mandatory investigation of fetal death^18^. However, even though this criterion defines fetal viability in relation to the available medical technology, the country uses the criterion of 20 weeks or more of gestation to make it mandatory to issue the DO and register the fetal death in the SIM^18^.

In the context of fetal viability, the more premature the fetus, the greater the risk of fetal death and the lower the reduction in fetal mortality in Brazil^46^. Longer pregnancies and those close to term had lower rates and greater reductions^47^. Prematurity and low birth weight were the main causes of fetal deaths, accounting for 73% of all stillbirths in Brazil between 1996 and 2021. In the period analyzed, these fetal deaths in premature babies increased by 2.41% and in low birth weight by 4.48%, data that corroborates previous studies^29^.

The vast majority (84%) of premature stillbirths had a low birth weight (< 2,500 g). Among term stillbirths, more than a third (37%) had low birth weight. This indicates that low birth weight is a significant risk factor for fetal death, even in term pregnancies^48^. Only a small minority of stillbirths (1%), both preterm and term, had high birth weight (macrosomia), suggesting that excess weight is not as important a risk factor as low birth weight for fetal death^46^.

It is essential to define strategies to reduce avoidable fetal deaths, with a focus on preventing prematurity and low birth weight^49,50^ through adequate care during pregnancy and childbirth^51^. Premature and low birth weight babies have a major impact on the increase in Fetal and Infant Mortality Rates. Strategies to reduce prematurity and low birth weight can have a significant impact on reducing these mortalities^52^.

The main causes of fetal deaths should be the focus of health policies and actions to reduce these FMR and the maternal and infant mortality ratio^52^. The interventions recommended before and during pregnancy to help reduce the risk of prematurity and low birth weight^51^ should address the main flaws in the control of these perinatal complications, which are related to the quality of prenatal care, the diagnosis of gestational alterations and obstetric management^53^.

The classification of stillbirths by the time of death in relation to childbirth was another indicator that pointed to the need to define specific interventions focused on the different stages of the pregnancy cycle, especially during pregnancy, in order to reduce fetal mortality. Most stillbirths (93%) occurred before delivery and a small proportion during delivery (6%). Furthermore, antepartum stillbirths increased by 22% and during stillbirths decreased by 74.52%.

This evidence points to the need to invest in actions that improve prenatal care, in order to reduce deaths before childbirth, and in improvements in childbirth care, in order to reduce deaths during pregnancy and childbirth^54^. On the other hand, the significant increase in antepartum deaths indicates shortcomings in care that need to be corrected in order to increase the chances of survival and further reduce preventable fetal deaths^55^.

Other studies show that fetal death is the most common adverse condition in pregnancy in Brazil and worldwide and is determined by multiple factors. These include obstetric factors, related to maternal health during pregnancy and childbirth; socioeconomic factors, such as the pregnant woman’s income, living conditions, and diet; environmental factors, such as exposure to risks during pregnancy^44^; sociodemographic factors, such as age, schooling, and other maternal data^58^; and biological factors, such as the mother’s and fetus’ previous health and genetic conditions^29^.

The covid-19 pandemic has intensified these multiple factors associated with fetal complications^15^ increasing deaths and halting the downward trend by accentuating pre-existing vulnerabilities in the Brazilian health system^151^. During the covid-19 pandemic (2020–2021), the cumulative increases in FMR were: 7.03% in the biennium for ≥ 20 weeks; 6.16% for ≥ 22 weeks; and 6.12% for ≥ 28 weeks, which may be related to factors such as the impact of the pandemic on access to and quality of health services, as well as possible direct effects of covid-19 infection on pregnant women^59^.

Analysis of the disparities and risk groups identified when stratifying FMR in Brazil according to sociodemographic and obstetric characteristics corroborates the influence of these various factors^49^. In this sense, the disparities observed in FMR in the country exemplify how multiple determinants interact in a complex way and influence the occurrence and trend patterns of fetal death in the country^44,48^. These data reveal the need to improve the quality of obstetric and prenatal care, as well as the economic and environmental conditions that affect pregnant women and fetuses^12^.

When considering these multiple factors in fetal mortality at GA ≥ 22 and ≥ 28 weeks, higher FMRs are observed among male conceptuses, showing the influence of biological factors^60^. Higher rates found at the extremes of maternal age, such as in adolescents aged 10 to 14 and women aged ≥ 45 years, reflect the impact of sociodemographic factors^61^. Higher FMRs among women with no schooling are indicators of socioeconomic disadvantage^62^. Home births, twin and triplet pregnancies, as well as vaginal births, exhibit higher FMRs, illustrating the relevance of obstetric factors^44^.

The risk groups compromising the reduction in fetal mortality, considering ≥ 22 and ≥ 28 weeks, included: adolescent mothers (10 to 19 years) and older mothers (45 to 49 years) with the lowest annual reduction rate, women with no schooling and low schooling (one to three years) with an annual increase rate, deliveries in non-hospital and home health facilities with an annual increase rate, premature pregnancies (less than 32 weeks, especially those less than 28 weeks), and low fetal weight (less than 2.500 g, especially less than 1,000 g), with a lower annual reduction rate and annual increase rate.

The groups made up of very young adolescents, women with no formal education, and home births reflect social inequalities and are generally associated with greater social vulnerability and less access to adequate prenatal care and qualified assistance during childbirth^63^. Extreme maternal ages (adolescents and women over 45), as well as multiple pregnancies (twins and triplets), prematurity, and low birth weight, represent biological risk factors that increase the chances of pregnancy complications, resulting in higher rates of fetal and maternal mortality^64,65^.

To solve the problems faced by the risk groups identified, some specific actions can be implemented: expanding access to family planning services and comprehensive sex education, especially for adolescents, in order to prevent early and unplanned pregnancies^66^; strengthening prenatal care programs, ensuring regular appointments, exams, and adequate guidance for all pregnant women, with special attention to risk groups^67^; promoting intersectoral partnerships and the integration of health, education, and social assistance services to address the underlying social determinants of fetal and maternal mortality^68^.

Specific actions should promote awareness and education on reproductive health, emphasizing the importance of institutional delivery and adequate prenatal care, particularly for women with no or little schooling^67^. It is crucial to improve the infrastructure and training of health professionals in health facilities, especially in remote or disadvantaged areas, in order to provide quality obstetric care^68^. In addition, implement social and economic support policies for vulnerable pregnant women, such as cash transfer programs and food assistance, in order to improve their living conditions and nutrition during pregnancy^68,69^.

The coordinated and comprehensive implementation of these actions can contribute to reducing risks and improving outcomes for vulnerable groups, mitigating inequalities and promoting equity in maternal and child health^68^. Neglecting the groups most at risk tends to diminish the impact of initiatives to reduce fetal mortality and perpetuate inequalities in fetal mortality.^67^. Thus, fetal deaths represent a significant public health challenge that needs to be addressed in order to further reduce FMR in the country^70^.

As a reflection of these significant challenges in health conditions, obstetric care and social and economic determinants that need to be addressed through intersectoral actions and specific public policies to improve indicators, most fetal deaths are linked to preventable causes, such as: prematurity, low birth weight, complications during pregnancy and childbirth, and socioeconomic factors. Potentially, the majority of fetal deaths in Brazil can be related to the poor quality of care provided during pregnancy and childbirth^71^.

In this context, progress in reducing fetal mortality rates in Brazil between 1996 and 2021 was influenced by different factors and causes related to pregnancy and childbirth, which still represent a high risk of fetal and perinatal death^12,72^, which are mainly associated with perinatal conditions, accounting for 94% of the causes of fetal deaths, especially maternal factors, pregnancy complications, intrauterine hypoxia, and birth asphyxia in the period analyzed. Congenital malformations accounted for 5% of cases.

Considering the list of deaths from preventable causes, approximately 67% of fetal deaths were potentially preventable, 39% of which could be reduced with improvements in Primary Health Care and 28% preventable with qualified hospital care during childbirth^73^. These data reflect the need for investment in ongoing efforts to improve prenatal and childbirth care in order to prevent potentially avoidable stillbirths and continue progress in fetal and infant survival^7,10^.

Stillbirths are often neglected in discussions, planning, and health systems^10^. This neglect is evident when comparing progress in reducing stillbirths with that of early neonatal deaths, where there has been more success (48% reduction). Stillbirths are largely absent from global data monitoring^74^. In addition, in several countries, quality data on stillbirths is not regularly produced, making it difficult to identify their causes^31^.

In addition to reflecting this negligence, Brazil’s fetal mortality seems to be a reflection of the focus on improving the quality of care for children under five and women, the focus on strengthening obstetrics^75^, the expansion of quality services for small and sick newborns^78^, and the reduction of inequalities^79^. However, while these interventions contributed to a greater reduction in the risk of neonatal, infant, and maternal death, which improved the health of children under five and women, and also contributed to a reduction in fetal mortality and equity in access to maternal and child health, they did not have the desired impact on perinatal health^80^.

It can be seen at a global level that the reduction in FMR, at GA ≥ 28 weeks, was 2.0% between 2000 and 2021, also slightly more than half the annual rate of reduction of 3.9% in infant mortality in the age group of one to 59 months in the same time interval^9^. These data highlight the need for additional efforts to accelerate the reduction of fetal mortality, with a view to achieving declines closer to those observed in infant mortality^7^.

It is noticeable that fetal mortality did not receive due attention in major global health initiatives until recently, when it was more emphatically addressed by the WHO^7^. In contrast to infant and maternal mortality, fetal mortality was not incorporated into major global health campaigns until 2014. It was not included as an indicator in the Millennium Development Goals (MDGs) and Sustainable Development Goals (SDGs)^81^. The WHO has only intensified the fight against preventable deaths, including fetal deaths, since 2014, through ENAP^82^ and the Global Strategy for Women’s, Children’s and Adolescents’ Health (2016–2030)^83^.

This global scenario reveals that fetal mortality has been underestimated in maternal and child health policies^11,49^. Globally, there are significant gaps in information related to fetal mortality, which makes it difficult to fully understand the problem and formulate policies and interventions^29,30^. Specifically, policies and investments have focused mainly on interventions and strengthening care during birth and the first week of life of the newborn, leaving the fetal phase neglected^75^.

However, despite being neglected and the possible impacts of the pandemic on maternal and child health care, the continued reduction in fetal mortality in Brazil and worldwide is a positive result^31^. This evidence indicates that public maternal and child health policies remain effective, even if they do not focus directly on reducing fetal mortality^84^. The gains in reducing the rates of this indicator before the pandemic, between 1996 and 2019, were sufficient to mitigate the increase in rates during the pandemic, in 2020 and 2021.

The long-term downward trend in FMR was not interrupted by the covid-19 pandemic, although there was a slowdown in the reduction compared to the pre-pandemic period. By maintaining the downward trend in FMR, with only a slowdown in the covid-19 pandemic interval, we can infer progress in comprehensive maternal and child care^85,86^ and highlight a possible reflection of the improvement in the quality of maternal and child care, with a focus on strengthening obstetrics and perinatal care, expanding quality services and reducing inequalities^78^.

The various interventions have contributed to reducing infant, maternal, and fetal mortality, improving the health of women and children and promoting equity in access to maternal and child health^80^. However, it is worth noting that the evidence points to the need for greater attention and investment, with a focus on comprehensive public policies, adequate data collection and analysis, and interventions aimed at the health and well-being of pregnant women and fetuses, including children under the age of five^12^.

There has been an improvement in filling in information on GA and birth weight in the stillbirth register, although this has not yet reached fully adequate levels^38^. In 2021, the percentage of ignored data on GA was approximately 7% for stillbirths, while ignored data on birth weight corresponded to around 6%.

There is a need to strengthen health information systems for monitoring and investigating preventable fetal deaths^87^. The investigation of fetal deaths, which began in Brazil in 2006, reduced uninvestigated deaths by 62% between 1996 and 2021. In the same period, there was an 80% decrease in deaths investigated without a summary form, and a 78% increase in deaths investigated with a summary form.

These evidence shows that it is crucial to invest in public policies for reproductive planning, adequate prenatal care and specialized care for high-risk, premature, and low-birth-weight pregnant women^88^ and expand access to and the quality of obstetric care, including early diagnosis of complications and emergency care^31^. In particular, investments should be made in technologies for the early recognition of high-risk pregnancies and timely access to regionalized health services with qualified care^89^.

On the other hand, reducing fetal mortality requires greater efforts to increase public awareness, improve data collection, evaluate progress, and understand local public health priorities^51^. In this way, the key to reducing stillbirths and giving them visibility is linked to investments in health information systems and in research that generates evidence for improving policies and practices in women’s and children’s health care, especially in the area of perinatal health^12^.

The aim is to reduce fetal mortality and mitigate the impact of factors that affect fetal mortality on child mortality, including maternal mortality. It has been shown that, although the long-term downward trend has slowed during the covid-19 pandemic, continued investment in policies and actions aimed at prevention, prenatal and childbirth care, as well as reducing socioeconomic inequalities and improving access to education, are key to meeting this challenge and promoting health equity.

### Regional Differences

The evidence by Major Region highlights regional inequalities in fetal mortality in Brazil between 1996 and 2021. There are significant differences between the regions, both in the absolute number of stillbirths and in the FMR per 1,000 total births. The Southeast and Northeast regions concentrated the highest absolute number of stillbirths, 39% and 29%, respectively, probably due to their higher population and number of births. However, the North (10.9/1,000) and Northeast (12/1,000) regions had the highest FMRs, suggesting a higher risk of fetal death in these locations.

The highest FMRs in the North and Northeast regions, with the worst socio-economic indices^90^ and lower access to prenatal and childbirth care^88^, may indicate inequalities in access to and quality of maternal and child health services, as well as socioeconomic inequalities^58^. These evidence reinforces the need to address regional disparities in order to reduce inequalities in fetal mortality throughout the country^91^.

The South, Southeast, and Center-West regions, presumably with better socioeconomic conditions and greater access to health services, have lower FMRs compared to the North and Northeast regions. This evidence of regional differences reinforces the findings that fetal mortality is influenced by demographic and socioeconomic factors, access to health services, education and general living conditions^43^.

Standardized, the average annual FMR in the North (10.4 ± 0.9), Northeast (10.6 ± 1.0), and Southeast (10.5 ± 1.9) were similar to the national average (10.7 ± 1.0) in Brazil, and higher than the South (8.6 ± 1.6) and Central-West (9.7 ± 0.9). These data indicate that while some regions have improved, others face substantial challenges in reducing fetal mortality, even excluding population factors, reinforcing persistent social and regional inequalities^5^.

This scenario of regional disparities in fetal mortality in Brazil, even after taking into account population differences, calls for more research to understand the factors behind these inequalities, such as the quality of prenatal care, the conditions of births, and the role of the public and private health systems in each region^5^. It is essential to direct prevention and improvement efforts towards the areas most at risk, guaranteeing equitable access and quality care during pregnancy and at the time of delivery, with the aim of reducing rates^64^.

There was no significant reduction in the number of stillbirths or in the FMR in the major Brazilian regions during the period analyzed, with a non-expressive downward trend or stability in the risk of fetal mortality and occasional fluctuations. The Central-West region presented the most worrying situation, with a consistently stable FMR in recent years.

The unimpressive downward trend, especially in the North and Northeast regions, and the stability in the Central-West can be attributed to persistent challenges in social and economic indicators and deficiencies in access to and quality of prenatal health care and childbirth^5^. Despite efforts to implement a unified health system focused on primary care, improvements have not yet been significantly reflected in the reduction of fetal mortality^86^.

A steeper decrease in FMR was observed in the Southeast and South, compared to the North and Northeast. In the Southeast, the reduction in FMR was around twice as high as in the North and 18 times higher than in the Northeast. Similarly, in the South, the reduction in firearm-related mortality rates was approximately twice as high as in the North and 13 times higher than in the Northeast. There are significant regional inequalities in fetal mortality in Brazil.

As for fetal mortality, in the period that includes the years of the covid-19 pandemic (1996–2021), the Northeast region showed a long-term downward trend. Before the pandemic (1996–2019), this region registered stability. The Central-West region, on the other hand, showed a downward trend before the pandemic and stability in the period covering pandemic years, the opposite of what was observed in the Northeast. In the North, Southeast, and South, during the pandemic period, there was a slowdown in the reduction of fetal mortality compared to the previous period.

These trends in the period that includes the pandemic can be attributed to the intensification of factors such as socioeconomic inequalities and the capacity of local health systems to respond to the pandemic, as well as possible differences in the policies adopted by states and municipalities to tackle covid-19^15,33^. It is essential to address these inequalities to ensure equitable and quality care during pregnancy and childbirth, considering the specificities and needs of each region^7^.

It is essential to invest on several fronts to tackle the challenges related to fetal mortality. Firstly, it is necessary to improve primary care and prenatal services, with an emphasis on the most vulnerable regions^11^. In addition, it is crucial to strengthen policies that reduce socioeconomic inequalities and promote equitable access to health services^48^. Another fundamental aspect is to implement training and qualification programs for the professionals involved in maternal and child care, guaranteeing quality assistance^49^.

At the same time, it is essential to promote health education and raise awareness of the risk factors associated with fetal mortality, involving the population^92^. Finally, it is necessary to improve surveillance and monitoring systems for maternal and fetal health, in order to support more effective interventions targeted at the specific needs of each region^93^. These integrated and comprehensive measures are essential to tackle this serious public health problem.

Fetal mortality continues to be one of the most frequent adverse pregnancy outcomes in Brazil, with disparities persisting between regions. The lack of attention and urgency to solve the problem of stillbirths has consequences: progress in reducing it becomes slow, and the burden remains high, jeopardizing fetal and neonatal survival. Neglecting stillbirths undermines efforts to reduce preventable deaths of fetuses and newborns. Thus, monitoring early fetal deaths, especially preventable ones, requires greater attention and investment, and is fundamental to the process of preventing and controlling fetal and perinatal mortality.

## CONCLUSION

Fetal mortality in Brazil, both nationally and in the Major Regions, showed significant long-term downward trends during the covid-19 pre-pandemic period (1996–2019). When including the years 2020 and 2021, corresponding to the pandemic (1996–2021), this downward trend continued, apart from the Central-West region, where the trend was downward before the pandemic and stable when including it, and the Northeast region, which showed stability in the pre-pandemic period and a long-term downward trend in the total period analyzed.

Specifically, the covid-19 pandemic had an impact on the increase in FMR and slowed down the reduction in 2020 and 2021. The long-term downward trend has not been interrupted, except in the Central-West region. In the Major Regions, the heterogeneity in APC, AAPC, and the disparities in FMR reflected regional inequalities.
