# Supplementary material for: Space-time trends in fetal mortality in Brazil, 1996–2021
Source: Rev Saude Publica. 2025 Mar 31;59:e2. doi: 10.11606/s1518-8787.2025059006194 (PMC11967338; doi:10.11606/s1518-8787.2025059006194)
Supplement: Translation

# Translation: Tendência espaço-temporal da mortalidade fetal no Brasil, 1996–2021

**Authors:** João Batista Francalino da Rocha, Italla Maria Pinheiro Bezerra, Elyecleyde Katiane da Silva Oliveira, Aline Bergamini Effgen Sena, Francisco Naildo Cardoso Leitão, Luiz Carlos de Abreu

**Affiliations:** I Faculdade de Medicina do ABC. Centro Universitário. Programa de Pós-Graduação em Ciências da Saúde. Santo André, SP, Brasil; II Escola Superior de Ciências da Santa Casa de Misericórdia de Vitória. Programa de Pós-Graduação em Políticas Públicas e Desenvolvimento Local. Vitória, ES, Brasil; III Universidade Federal do Acre. Laboratório Multidisciplinar de Estudos e Escrita Científica em Ciências da Saúde. Rio Branco, AC, Brasil; IV Universidade Federal do Espírito Santo. Departamento de Educação Integrada em Saúde. Vitória, ES, Brasil; V Universidade Federal do Acre. Programa de Pós-Graduação em Ciências da Saúde. Rio Branco, AC, Brasil; VI Universidade Federal do Espírito Santo. Programa de Pós-Graduação em Saúde Coletiva. Vitória, ES, Brasil

**Keywords:** Mortalidade Perinatal, Mortalidade Fetal, Morte Fetal, Natimorto, Assistência Perinatal, COVID-19, Desigualdades da Saúde

## Abstract

**OBJETIVO:**

Avaliar a tendência espaço-temporal da mortalidade fetal no Brasil no período de 1996 a 2021.

**MÉTODOS:**

Estudo ecológico de séries temporais com dados secundários de mortes fetais com idade gestacional (IG) ≥ 20 semanas do Sistema de Informações sobre Mortalidade (SIM) no Brasil, entre 1996 e 2021, pré-pandemia de covid-19 (1996–2019) e anos de 2020 e 2021 da pandemia. Analisou a taxa de mortalidade fetal (TMF) para identificar o risco anual de morte fetal na população específica. Foram calculadas a variação percentual (VP), variação percentual anual (VPA) e variação percentual média anual (VPMA) por meio de regressão Joinpoint, para determinar os padrões de tendência: crescente, decrescente ou estacionária. Utilizou-se os softwares Excel 2019, Stata e Joinpoint Regression.

**RESULTADOS:**

No Brasil, mortes fetais na IG ≥ 20 semanas representaram 1,14% dos nascimentos e 58% das mortes perinatais no período analisado. Cerca de 93% foram anteparto, 6% intraparto e 1% foram registradas como ocorrido após o parto. A TMF global do período, considerando a IG ≥ 20, ≥ 22 e ≥ 28 semanas, foi de 11,4, 10,7 e 8,6/1.000 nascimentos, respectivamente. Apesar da tendência crescente dos natimortos nas mortes perinatais, desaceleração na redução e aumento durante a pandemia de covid-19, a TMF, nas idades gestacionais avaliadas, diminuiu 20%, 25% e 41%, respectivamente, com VPMA de -1,00, -1,13 e -1,89.

**CONCLUSÃO:**

A mortalidade fetal apresentou tendência decrescente de longo prazo em âmbito nacional e regional no Brasil, com exceção da região Centro-Oeste, onde a tendência foi estacionária. A pandemia de covid-19 desacelerou a redução e aumentou a medida, no entanto, a tendência decrescente não foi interrompida. No âmbito regional, as TMF mais elevadas e as menores reduções ocorreram no Norte, Nordeste e Centro-Oeste, evidenciando desigualdades regionais.

## INTRODUÇÃO

A mortalidade fetal é um dos indicadores de saúde materno-infantil amplamente utilizados para analisar as condições de vida de uma população, uma vez que reflete a ocorrência de fatores vinculados à gestação e ao parto, condições de acesso a serviços de saúde e a qualidade da assistência prestada às mulheres durante a gravidez^1^. É um resultado adverso comum da gravidez, em sua maioria evitável com intervenções adequadas^4^, porém a prevenção e redução ainda são invisibilizadas nas ações programáticas e nos relatórios de monitorização de saúde em todo o mundo^2,4^.

Os natimortos não foram contabilizados nos Objetivos de Desenvolvimento do Milênio (ODM), nem monitorados pela Organização das Nações Unidas (ONU), nem computados nas métricas da Carga Global de Doenças, nem tiveram meta específica nos Objetivos de Desenvolvimento Sustentável (ODS)^5^. No entanto, as intervenções para a redução na mortalidade materna e em menores de cinco anos nos ODM, ajudaram a reduzir a mortalidade fetal, e essa tem recebido atenção crescente nas agendas mundiais e ações programáticas^6^.

Nos ODS, a meta 3.1 estabelece reduzir mortalidade materna global para menos de 70 mortes por 1.000 nascidos vivos e a 3.2, acabar com as mortes evitáveis de recém-nascidos e crianças menores de cinco anos, reduzindo a mortalidade neonatal para pelo menos 12 por 1.000 nascidos vivos e a mortalidade de crianças menores de cinco anos para pelo menos 25 por 1.000 nascidos vivos até 2030. Embora não haja uma meta específica para a mortalidade fetal, sua redução é fundamental para atingir essas metas^7^.

A Estratégia Global para a Saúde da Mulher, da Criança e do Adolescente (2016–2030) da Organização Mundial de Saúde (OMS) e o Plano de Ação para Todos os Recém-Nascidos (Enap) liderado pelo Fundo das Nações Unidas para a Infância (Unicef) e pela OMS incluem ações para prevenir a mortalidade materna e infantil, incluindo a redução da mortalidade fetal por meio do fortalecimento dos serviços de saúde materna e neonatal^7^.

A Organização Pan-Americana da Saúde (Opas) tem apoiado os países da região nas ações de vigilância da mortalidade fetal, capacitação de profissionais e fortalecimento dos sistemas de informação em saúde para monitorar esse indicador^8^. Muitos países têm incorporado metas e ações para reduzir a mortalidade fetal em seus planos e políticas de saúde materna e infantil, como o Plano Nacional de Prevenção da Mortalidade Materna e Infantil no Brasil^6^.

Globalmente, houve uma redução de 35% na taxa de natimortalidade entre 2000 e 2021, entre fetos com idade gestacional (IG) ≥ 28 semanas, que diminuiu de 21,3 natimortos por 1.000 nascimentos totais em 2000 para 13,9 em 2021, com taxa de redução anual de 2,0^9,10^. Embora algum progresso ter sido feito, a mortalidade fetal continua sendo um problema global^11^ com cerca de 2 milhões de natimortos por ano nos últimos anos, resultando em uma perda tremenda de 53 milhões de natimortos desde o ano 2000^12^.

Na América Latina e no Caribe, as estimativas apontam uma redução de 31% na taxa de natimortos (IG ≥ 28 semanas), saindo de 11,2 natimortos por 1.000 nascimentos totais para 7,74, nesse mesmo período^9,10^. No Brasil, a taxa de natimortalidade na IG ≥ 28 semanas passou de 10,12 natimortos por 1.000 nascimentos totais, em 2000, para 6,98 nascimentos totais, em 2015^9^. Contados os óbitos perinatais, considerando o critério nacional de 22 semanas de gestação até seis dias de vida, 96,62% das mortes perinatais se enquadraram como natimortos^13^. Pelo critério da OMS de 28 semanas de gestação até seis dias, essa proporção foi de 85,53% no país^10^.

O Brasil é um país com grandes desigualdades socioeconômicas e regionais, o que pode influenciar diretamente os indicadores de saúde materno-infantil, incluindo a mortalidade fetal. Fatores como o baixo nível de escolaridade materna, más condições de moradia e saneamento, dificuldade de acesso aos serviços de saúde e baixa qualidade da assistência pré-natal tendem a ser mais prevalentes nas regiões mais pobres, como o Norte e Nordeste^14^.

Cerca de 73% dos natimortos têm como causas principais a prematuridade e o baixo peso ao nascer (< 2.500 gramas)^7^. Quase a metade de todos os natimortos acontece durante o trabalho de parto e o nascimento^2,4^. Dessa forma, a maioria dos óbitos decorre de problemas que podem ser evitados com atendimento materno e fetal adequado, como prevenção ou tratamento de infecções e complicações da gravidez (por exemplo, hipertensão arterial, diabetes e retardo do crescimento do feto antes do nascimento)^5,11^.

Entretanto, além disso, sua redução requer acesso equitativo a cuidados perinatais de qualidade, educação materna, capacitação profissional e melhoria das condições socioeconômicas^2^, e o monitoramento e a análise contínua dos dados de mortalidade perinatal são cruciais para implementar políticas efetivas de redução^8^. A pandemia de covid-19 impôs desafios adicionais a esse cenário para a saúde fetal tanto no âmbito local quanto global, os quais precisam ser quantificados^15,16^.

Neste estudo, o objetivo foi avaliar a tendência espaço-temporal da mortalidade fetal no Brasil no período de 1996 a 2021. Este intervalo temporal compreende um período pré-pandemia (1996–2019) e os anos 2020 e 2021 da pandemia de covid-19. A hipótese era de que a mortalidade fetal, tanto em nível nacional, no Brasil, quanto regional, em suas Grandes Regiões, apresentaria tendências decrescentes de longo prazo significativas ao longo do tempo, no período pré-pandemia, e incluindo a pandemia de covid-19 (1996–2021).

Estabeleceram-se três objetivos específicos: 1) identificar os padrões de tendências nas taxas de mortalidade fetal em nível nacional e regional ao longo do período analisado; 2) identificar padrões e variações geográficas na mortalidade fetal entre Grandes Regiões durante o mesmo período; 3) descrever os padrões de risco e de tendência, por fatores demográficos e obstétricos, bem como por causas de mortes nos índices de mortalidade fetal em nível nacional no período em análise. Esses dados e informações podem subsidiar maior atenção e ações direcionadas para reduzir, de forma mais rápida e eficaz, as mortes fetais evitáveis.

## MÉTODOS

### Concepção e População do Estudo

Estudo ecológico de séries temporais dos índices anuais de mortalidade fetal no Brasil entre 1996 e 2021, compreendendo o período pré-pandêmico (1996–2019) e os anos de 2020 e 2021 da pandemia de covid-19. Utilizou-se dados secundários referentes ao registro de óbitos fetais com IG ≥ 20 semanas do Sistema de Informação sobre Mortalidade (SIM) do Departamento de Informática do Sistema Único de Saúde (Datasus) do Ministério da Saúde (MS) do Brasil^17^.

Estimou-se as variações percentuais anual e média anual para determinar os padrões de tendências para as medidas absolutas, número de natimortos, e relativas, proporções e taxas de mortalidade fetal, tanto para o Brasil quanto para suas Grandes Regiões (Norte, Nordeste, Sudeste, Sul e Centro-Oeste). Para essas estimativas, foram considerados os natimortos nos estratos de IG ≥ 20, ≥ 22 e ≥ 28 semanas.

No âmbito nacional, a população do estudo foi composta por 886.878 registros de óbitos fetais com IG ≥ 20 semanas. No âmbito regional, a população do estudo foi composta por esses registros de óbitos fetais, de acordo com o registro por Grande Região brasileira, que totalizou 886.852 casos (26 casos a menos em relação ao total nacional), o correspondente a 92.613 casos (10,44%) na região Norte, 285.896 casos (32,24%) na região Nordeste, 350.546 casos (39,53%) na região Sudeste, 97.187 casos (10,96%) na região Sul e 60.610 casos (6,83%) na região Centro-Oeste, no período analisado.

A população de natimortos em nível nacional, foi desagregada em três estratos de idade gestacional: IG ≥ 20 semanas (100% dos registros); IG ≥ 22 semanas, 835.153 casos (94% dos registros); e IG ≥ 28 semanas, 665.287 casos (75% dos registros). Em nível regional, foi estratificada em: IG ≥ 20 semanas, conforme distribuição citada anteriormente; e IG ≥ 22 semanas, 835.130 casos, distribuídos por Grande Região da seguinte forma: 86.916 casos (10,41%) na Norte, 270.825 casos (32,43%) na Nordeste, 327.118 casos (39,17%) na Sudeste, 92.900 casos (11,12%) na Sul e 57.371 casos (6,87%) na Centro-Oeste.

Essa estratificação da população de natimortos, no âmbito nacional e regional, foi feita para permitir analisar os óbitos fetais: 1) quanto ao registro a partir da IG ≥ 20 semanas, normatizado pelo MS do Brasil com emissão da Declaração de Óbito (DO) e registro no SIM^18,19^; 2) quanto a classificação de natimortos de IG ≥ 22 semanas, preconizada pela OMS e adotada na CID-10^20^, e IG obrigatória de investigação do óbito fetal normatizada pelo MS^13^ e comparações dentro do país; 3) quanto a IG ≥ 28 semanas, classificação internacional que permite comparações entre países e regiões globais.

Para permitir o cálculo das taxas de mortalidade fetal (TMF), adotou-se a população anual de nascimentos totais (NT), que totalizou 77.910.333 registros, média anual de 2.910.333, no período de 1996 a 2021. Esta foi composta por 77.075.180 registros de nascidos vivos do Sistema de Informação sobre Nascidos Vivos (SINASC) e os registros de natimortos. Destaca-se que os NT foram também estruturados em três estratos, considerando a IG: ≥ 20; ≥ 22; e ≥ 28 semanas dos natimortos.

### Coleta de Dados e Procedimentos

Os dados sobre natimortos foram coletados do SIM e nascidos vivos do SINASC, tabulados por meio do TabNet *on-line*. O TabNet é uma ferramenta de tabulação desenvolvida pelo Datasus do MS brasileiro, que permite a realização de tabulações *on-line* de dados e a geração de planilhas a partir da base de dados do Sistema Único de Saúde (SUS) do Brasil. Trata-se de um tabulador de domínio público, disponível no *site* https://datasus.saude.gov.br/informacoes-de-saude-tabnet/. O acesso aos dados foi realizado nos dias 7 e 8 de janeiro de 2022^17^.

Os passos seguidos na extração dos dados sobre natimortos foram: no TabNet, acessou-se “Estatísticas Vitais”, “Mortalidade – desde 1996 pela CID-10”, “Óbitos fetais”, área de abrangência “Brasil por Região e Unidade da Federação”. No ambiente de tabulação, selecionou-se no campo “Coluna” o ano do óbito; no campo “Conteúdo”, o óbito por residência; nos “Períodos disponíveis”, as datas que atendiam ao estudo; no campo “Seleções disponíveis”, na duração da gestação, considerou-se três estratos de IG: ≥ 20, ≥ 22 e ≥ 28 semanas, mais a categoria “ignorado”; por fim, no campo “Linha”, selecionou-se uma por vez as variáveis de interesse do estudo^17^.

Para a extração dos dados sobre nascidos vivos, seguiu-se os passos: no TabNet, acessou-se “Estatísticas Vitais”, “Nascidos vivos – desde 1994”, selecionou-se “Nascidos vivos”, área de abrangência “Brasil por Região e Unidade da Federação”. No ambiente de tabulação, selecionou-se no campo “Coluna” o ano de nascimento; no campo “Conteúdo”, o nascimento por residência da mãe; nos “Períodos disponíveis”, as datas que atendiam ao estudo; e no campo “Linha”, uma por vez, as variáveis de interesse para a pesquisa^17^.

O desfecho investigado foi o padrão de tendência dos índices de mortalidade fetal no Brasil ao longo do tempo, no âmbito nacional e regional. Buscou-se identificar se esses índices eram crescentes, decrescentes ou estacionários. Para tanto, estimou-se a variação percentual (VP), a variação percentual anual (VPA) e a variação percentual média anual (VPMA).

A variável preditora, é o “tempo”, expresso em anos-calendário, que foi utilizada para modelar a evolução dos índices de mortalidade fetal. Os índices de mortalidade fetal foram explorados por características, agrupadas em: sociodemográficas e obstétricas e por capítulo e lista de causas de morte segundo a Classificação Internacional de Doenças (CID-10).

O grupo de variáveis sociodemográficas foi composto por: sexo do natimorto (masculino e feminino); idade materna em anos, categorizada em classes de faixa etária com intervalos de cinco anos cada, iniciando em 10–14 até 49 anos e mais; escolaridade materna em anos completos de estudo da gestante no momento do parto, categorizada em: nenhum, 1–3 anos, 4–7 anos, 8–11 anos e 12 anos ou mais.

O grupo de variáveis obstétricas incluiu: duração da gravidez em semanas, categorizada em < 22 semanas e, para IG ≥ 22 semanas, foi distribuída em categorias com intervalos de cinco semanas até 42 semanas e mais; o peso ao nascer em gramas (g), categorizado em < 500 g, e a partir de 500 g, distribuído em intervalos de 500 g até 2.500–2.999 g, 3.000–3.999 g e 4.000 g e mais; tipo de gravidez (única, dupla e tripla e mais); e tipo de parto (cesáreo e vaginal).

A mortalidade fetal foi ainda analisada em relação local de ocorrência (hospital, outro estabelecimento de saúde, domicílio, outros locais como vias públicas e aldeias indígenas); momento do óbito fetal em relação ao parto (antes, durante e após); causa básica do óbito fetal categorizada por capítulos e lista de causas; e Grandes Regiões: Norte, Nordeste, Sudeste, Sul e Centro-Oeste do Brasil.

### Análise Estatística

As TMF foram calculadas para os estratos de IG ≥ 20, ≥ 22 e ≥ 28 semanas. No cálculo da TMF, levando-se em consideração a subnotificação de óbitos fetais e a precariedade das informações sobre a duração da gestação, foram somados ao numerador e denominador o número de óbitos fetais com IG ignorada ou não preenchida. O método de cálculo utilizado foi o direto, aplicando-se a seguinte fórmula:

coeficiente de mortalidade fetal ou taxa mortalidade fetal, método de cálculo.


$$\text{ TMF }=\frac{\begin{array}{c} \text{ Número de óbitos fetais (22 semanas de }\\ \text{ gestação e mais), de mães residentes } \end{array}}{\text{ Número de nascimentos totais de mães }}\times 1.000$$ (1)

A análise explicativa dos padrões de tendência das séries temporais da mortalidade fetal foi realizada por meio da construção de modelos de regressão não segmentada e segmentada dos índices desse indicador, utilizando o programa *Joinpoint Regression Analysis* ^21^. Em outras palavras, foi conduzida tanto a análise de tendência de longo prazo quanto a análise de tendência interrompida, verificando se uma linha com um ponto ou múltiplos pontos de inflexão era estatisticamente significante, considerado alfa = 0,05.

Na análise de tendência interrompida (segmentada) na série temporal dos índices do indicador analisado, a seleção do número de pontos de inflexão para obter o modelo significativo foi realizada utilizando-se as configurações padrão do *software* pelo método *Grid Search* (que permite que os pontos de inflexão ocorram exatamente nos anos observados).

Os testes de significância estatística para escolha/avaliação do melhor modelo da regressão basearam-se no método de permutação de Monte Carlo^22,23^, especialmente útil considerando o conjunto de dados analisados, em que os pressupostos dos testes paramétricos (como normalidade ou homocedasticidade) são violados e a distribuição teórica do teste estatístico é difícil de calcular^22,23^.

Para a análise dos padrões de tendência no *software Joinpoint Regression*, na modelagem, definiu-se a frequência absoluta, e a proporção dos natimortos e a TMF por categoria das variáveis do estudo como desfechos, e os anos de ocorrência desses indicadores como variável preditora.

O *software Joinpoint Regression Analysis* ^21^ foi configurado para calcular a VPMA nos seguintes períodos: 1996–2019 – anterior à pandemia de covid-19; e 1996–2021 – intervalo incluindo os anos da pandemia de covid-19. Adicionalmente, nos casos em que as linhas apresentaram um ou mais pontos de inflexão, foi calculada a VPA para cada segmento^22,23^.

Para os casos de tendência sem segmento, a VPMA é igual à VPA. A tendência foi considerada crescente para VPMA e VPA que apresentaram valores positivos e significativamente diferente de zero. A tendência foi considerada decrescente quando esses valores foram negativos e significativamente diferentes de zero. Quando os valores negativos ou positivos não foram significativamente diferentes de zero, a tendência foi classificada como estacionária. Tanto para a análise da VPAM quanto da VPA, foram considerados um nível de significância estatística menor que 0,05 (p < 0,05) e intervalos de 95% de confiança^22,23^.

Como o modelo de regressão por ponto de inflexão assume uma tendência linear entre os pontos, com os mesmos pressupostos da regressão linear, exceto o de homoscedasticidade e não autocorrelação, foram incorporados na configuração ao *software Joinpoint*, ou seja, ajustou-se um modelo de regressão ponderado^23^. Para tanto, selecionou-se a opção autocorrelacionado de primeira ordem estimada a partir dos dados. Dessa forma, o programa assumiu que os erros aleatórios eram autocorrelacionados e estimou os coeficientes de regressão por mínimos quadrados ponderados^22^.

Para validar o modelo de regressão, foram realizados testes para verificar três pressupostos importantes: normalidade, homocedasticidade e não autocorrelação dos resíduos.

Para evidenciar a distribuição não normal dos resíduos, foram realizados os testes de normalidades de *Kolmogorov-Sminorv* e de *Shapiro-Wilk*, com p-valor < 0,05 em ambos os testes. Concluiu-se pela não normalidade se p < 0,05, rejeitando a hipótese nula. Se p > 0,05, não se rejeitava a normalidade, indicando que os dados poderiam ser considerados normalmente distribuídos^24^.

A homocedasticidade, que pressupõe que a variância dos resíduos é constante, foi verificada pelo teste de *Breusch-Pagan*, que testa a hipótese nula de homocedasticidade contra formas alternativas de heterocedasticidade. Teve-se como hipótese nula a homocedasticidade, em que para valores de p < 0,05, a homocedasticidade é rejeitada ao nível de significância de 5%. O pressuposto de não autocorrelação dos resíduos, ou seja, que eles são independentes ao longo do tempo, foi avaliado pelo teste de *Durbin-Watson*, que verifica a existência de autocorrelação de primeira ordem^25^.

Para analisar os padrões temporais dos dados estatísticos, utilizou-se ainda o cálculo da VP, feito ano a ano, considerando ano posterior e o anterior, e VP entre o ano final e o ano inicial da série temporal, amplamente empregados nesse tipo de análise. Em outras palavras, calculou-se a amplitude da variação ocorrida no período de 1996 a 2021, bem como entre cada par de anos consecutivos desse período. O resultado foi expresso em percentagem, por meio da seguinte fórmula:

Variação percentual, método de cálculo:


VP=[( valor no momento posterior ÷ valor no momento anterior )−1]×100 (2)

Dessa forma, a VP, a VPA para diferentes segmentos de reta e a VPMA por um segmento de reta foram as medidas utilizadas para analisar as mudanças e tendências de aumento, redução e estabilidade nos índices dos indicadores avaliados. Foram empregados na análise estatística o aplicativo *Microsoft Office Excel* versão 2019, o *software Statistics and Data Science* (STATA), versão 17, e o *software Joinpoint Regression,* versão 5.10.

### Limitações, Problemas Operacionais e Formas de Minimizá-los

Uma das limitações para o cálculo das TMF decorre do fato do registro de óbitos fetais ser provenientes de diferentes fontes, o que pode implicar qualidade variável da informação e completude variada dos registros no SIM e SINASC. Em um sistema de informações, a completude refere-se ao grau de preenchimento de cada campo analisado, mensurada pela proporção entre campos preenchidos e não preenchidos^26^.

A completude dos dados é um importante indicador de qualidade, uma vez que campos incompletos ou não preenchidos podem comprometer a confiabilidade e a acurácia das análises realizadas com base nessas informações^27^. A não completude dos dados, caracterizada por registros incompletos, sem preenchimento ou preenchidos com a opção “ignorado” referente às variáveis sociodemográficas, obstétricas e causas de natimortos, impactou os resultados obtidos.

A inclusão ou exclusão dessas variáveis nas análises foi realizada conforme a seguinte classificação da completude dos dados: excelente (acima de 95% de completude); boa (de 90 a 95%); regular (de 80 a 90%); ruim (de 50 a 80%); e muito ruim (abaixo de 50%)^27^. Variáveis com completude abaixo de 50% foram excluídas das avaliações. Alguns dados da DO fetal não foram registrados, entre eles destacou-se os da variável raça/cor que são essenciais para orientação de intervenções preventivas.

Nesse caso, a variável “raça/cor” apresentou alta prevalência de informações não preenchidas e, portanto, foi excluída das análises subsequentes. Nessas, a incompletude dos dados variou de 1,82% para a variável “local de ocorrência” a 95,8% para a variável “raça/cor”. A categoria “causas mal definidas” referente ao Capítulo XVI da CID-10 ultrapassou 29% como registro de informação incompleta, o que compromete as estatísticas de mortalidade fetal^26^. A ausência de quase 96% dos registros de uma variável crítica como raça/cor é excepcionalmente alta e poderia ter introduzido um viés substancial nas análises estatísticas.

Nas demais variáveis incluídas neste estudo, o percentual de dado “ignorados” ou não preenchido variou de 1,82% a 32,95% (instrução materna). No entanto, com o objetivo de minimizar vieses decorrentes de registros não preenchidos, empregou-se a imputação dos dados faltantes. Para tanto, utilizou-se o método de imputação por distribuição proporcional, adotado pelo Instituto Brasileiro de Geografia e Estatística (IBGE), que consistiu no agrupamento dos casos com informação ignorada ou não declarada, seguido do cálculo das proporções das categorias conhecidas. Os casos com informação ignorada foram redistribuídos proporcionalmente, preservando-se as proporções originais das categorias válidas^28^.

Cautelarmente, considerando-se limitações associadas à imputação, foram realizadas análises de sensibilidade para avaliar o impacto da imputação nos resultados principais. A análise de sensibilidade para avaliar o impacto da imputação nos resultados considerou se ambas as amostras tinham distribuições semelhantes. Para tanto, forma efetuados os testes de *Kolmogorov-Sminorv* e de *Shapiro-Wilk*, antes e depois da imputação^28^. Não foram encontrados p-valores abaixo do nível de significância (p = 0,05), concluindo-se que as distribuições não sofreram alterações após o procedimento de imputação.

Adicionalmente, na análise de sensibilidade, avaliou-se o impacto da imputação de valores faltantes nos índices e padrões de tendência de mortalidade fetal. Realizou-se um comparativo entre o índice antes da imputação e a nova série temporal recalculada após o procedimento de imputação. As informações revelaram que os novos resultados foram mais elevados do que os originais sem imputação, em todo o período analisado. No entanto, apresentaram uma trajetória que acompanhou a série temporal anterior e mesmo padrão de tendência.

### Aspectos Éticos e Legais

Este estudo atendeu aos preceitos éticos garantidos na Declaração de Helsinque e nas resoluções brasileiras n^o^ 466/12 e n^o^ 510/16 do Conselho Nacional de Saúde, que tratam da pesquisa em seres humanos.

Não foi necessária a submissão do projeto de estudo ao Comitê de Ética em Pesquisa (CEP) em Seres Humanos, uma vez que se trata de dados secundários, sem identificação dos participantes, conforme previsto na Resolução do Conselho Nacional de Saúde (CNS) n^o^ 466, de 12 de dezembro de 2012, e na Resolução CNS n^o^ 510, de 7 de abril de 2016. Esta última dispõe sobre a não necessidade de submissão a um CEP pesquisa para estudos que utilizem informações de domínio público.

## RESULTADOS

### Tendências Nacionais

O Brasil registrou, diariamente, uma média de 94 óbitos fetais, o que corresponde a aproximadamente 34.111 casos anuais, entre 1996 e 2021. Foram contabilizados 886.878 natimortos com IG igual ou superior a 20 semanas no país, representando uma taxa global de mortalidade fetal de 11,4 óbitos por 1.000 nascimentos totais. Desse total, 94% (835.153 casos) concentraram-se na IG ≥ 22 semanas, equivalente a uma TMF global de 10,7/1.000, e 75% (665.287 casos) ocorreram com IG ≥ 28 semanas, compondo uma TMF global de 8,6/1.000.

A média anual de natimortos, considerado os estratos de IG ≥ 20, ≥ 22 e ≥ 28 semanas, foi de 34.111, 32.121 e 25.588 com desvio-padrão de ±3.958, ±4.144 e ± 4.633, respectivamente, no período analisado. A variabilidade em torno da média anual de natimortos, nos mesmos estratos de IG, foi de 11,60%, 12,90% e 18,11%, respectivamente.

O número absoluto de natimortos diminuiu gradativamente, passando de 40.434, 39.892 e 37.823 casos, respectivamente, em 1996, para 29.325, 27.022 e 20.186 em 2021, considerando as IG ≥ 20, ≥ 22 e ≥ 28 semanas. A proporção anual de natimortos em relação ao total no período, nas IG analisadas, apresentou uma tendência decrescente ao longo dos anos, reduzindo de 4,56%, 4,78% e 5,69% em 1996 para 3,31%, 3,24% e 3.03% em 2021, respectivamente.

A taxa média anual de mortalidade fetal, para a IG ≥ 20 semanas, foi de 11,3 (±0,9 de desvio padrão). Para a IG ≥ 22 semanas, essa taxa foi de 10,7 (±1,0 DP), enquanto na IG ≥ 28 semanas, ela foi de 8,5 (±1,3 DP), enquanto para a IG ≥ 28 semanas, foi de 8,5 (±1,3 DP). Considerando a IG ≥ 20 semanas, a probabilidade de um feto nascer morto, a cada 1.000 nascimentos totais, foi 1,06 vezes maior do que a observada para a IG ≥ 22 semanas e 1,33 vezes maior do que a verificada para a IG ≥ 28 semanas. Por outro lado, a TMF média anual para a IG ≥ 22 semanas foi 1,25 vezes superior à observada para a IG ≥ 28 semanas.

As TMF anuais estimadas apontaram para uma redução do risco de morte fetal e um aumento da sobrevivência perinatal. A Figura apresenta as séries temporais das TMF estimadas, considerando os estratos de IG ≥ 20, ≥ 22 e ≥ 28 semanas, no Brasil, entre 1996 e 2021. Os dados indicam uma tendência de crescimento de longo prazo para as TMF, porém com flutuações e períodos distintos de aumento ou diminuição em segmentos específicos das séries temporais, variações essas que são apresentadas na Tabela 1.

**Figura:** Séries temporais de taxas de mortalidade fetal nos estratos de idade gestacional ≥ 20, ≥ 22 e ≥ 28 semanas. Brasil, 1996–2021.

IG: idade gestacional.

Fonte: baseado em dados do Ministério da Saúde e Sistema de Informações sobre Mortalidade, 2022.

**Tabela 1:** Análise de tendências de longo prazo e interrompida nas séries temporais de óbitos fetais e das taxas de mortalidade fetal nos estratos de idade gestacional ≥ 20, ≥ 22 e ≥ 28 semanas para antes e incluindo a pandemia de covid-19. Brasil, 1996–2021.

Variáveis	Segmento	VPA	IC95%	VPMA	IC95%	Interpretação
Óbitos fetais com idade gestacional ≥ 20 semanas	1996–1999	0,13	(-1,37 a 2,80)			Crescente
	1999–2008	-2,81^*^	(-3,48 a 2,50)			Crescente
	2008–2014	0,29	(-0,34 a 1,87)			Decrescente
	2014–2021	-1,60^*^	(-2,34 a -1,15)			Crescente
	1996–2019			-1,37^*^	(-1,49 a -1,22)	Decrescente
	1996–2021			-1,39^*^	(-1,52 a -1,25)	Decrescente
Óbitos fetais com idade gestacional ≥ 22 semanas	1996–2003	-1,50^*^	(-1,97 a -0,82)			Decrescente
	2003–2006	-4,29^*^	(-4,90 a -1,84)			Decrescente
	2006–2012	-1,39^*^	(-3,11 a -0,56)			Decrescente
	2012–2015	1,14	(-0,52 a 1,84)			Estacionária
	2015–2021	-1,98^*^	(-2,85 a -1,46)			Decrescente
	1996–2019			-1,58^*^	(-1,67 a -1,48)	Decrescente
	1996–2021			-1,61^*^	(-1,73 a -1,51)	Decrescente
Óbitos fetais com idade gestacional ≥ 28 semanas	1996–2008	-3,51^*^	(-3,96 a 3,15)			Decrescente
	2008–2014	0,59	(-0,51 a 3,60)			Estacionária
	2014–2021	-2,61^*^	(-4,55 a -1,76)			Decrescente
	1996–2019			-2,26^*^	(-2,42 a -2,10)	Decrescente
	1999–2021			-2,29^*^	(-2,50 a -2,12)	Decrescente
Taxa de mortalidade fetal na idade gestacional ≥ 20 semanas	1996–2007	-1,77^*^	(-3,10 a -1,27)			Decrescente
	2007–2021	-0,4	(-0,74 a 0,46)			Decrescente
	1996–2019			-1,06^*^	(-1,23 a -0,89)	Decrescente
	1996–2021			-1,00^*^	(-1,19 a -0,81)	Decrescente
Taxa de mortalidade fetal na idade gestacional ≥ 22 semanas	1996–1999	-3,99^*^	(-6,30 a -2,75)			Decrescente
	1999–2003	-0,28	(-1,12 a 1,03)			Estacionária
	2003–2006	-3,19^*^	(-3,85 a -1,95)			Decrescente
	2006–2019	-0,75^*^	(-1,03 a -0,56)			Decrescente
	2019–2021	2,23	(-0,01 a 3,66)			Estacionária
	1996–2019			-1,42^*^	(-1,53 a -1,28)	Decrescente
	1996–2021			-1,13^*^	(-1,29 a -1,01)	Decrescente
Taxa de mortalidade fetal na idade gestacional ≥ 28 semanas	1996–2000	-5,44^*^	(-10,11 a -2,51)			Decrescente
	2000–2007	-1,9	(-4,45 a 1,29)			Estacionária
	2007–2021	-0,85	(-5,32 a 1,94)			Estacionária
	1996–2019			-1,98^*^	(-2,23 a -1,61)	Decrescente
	1996–2021			-1,89^*^	(-2,27 a -1,53)	Decrescente

IC95%: intervalo de confiança de 95%.

Fonte: baseado em dados do Ministério da Saúde e do Sistema de Informação sobre Mortalidade, 2022.

Nota: *Indica que a variação percentual anual (VPA) e a variação percentual anual média (VPMA) são significativamente diferentes de zero no nível alfa = 0,05. A interpretação de tendência interrompida é dada pela VPA, e de tendência de longo prazo é pela VPMA.

A Tabela 1 apresenta a análise de tendências de longo prazo e de tendências interrompidas nas séries temporais de natimortos e das TMF nos estratos de IG ≥ 20, ≥ 22 e ≥ 28 semanas, no Brasil, entre os anos de 1996 e 2021. A análise dessas tendências é realizada por meio do cálculo da VPA e da VPMA.

O número absoluto de natimortos na IG ≥ 20 semanas apresentou redução de 28% (de 40.434 em 1996 para 29.325 em 2021), com VPMA de -1,39. A redução foi mais acentuada para a IG ≥ 22 semanas, diminuindo 32% (VPMA -1,62; de 39.892 para 27.022), e para a IG ≥ 28 semanas, com uma queda de 47% (VPMA -2,29; de 37.823 para 20.186). Na IG ≥ 20 semanas, destacaram-se os segmentos de 1996 a 2008 e de 2014 a 2021, com tendência crescente. Já para a IG ≥ 22 semanas, observou-se um período de estabilidade entre 2012 e 2015, seguido por um decréscimo de 2015 a 2021. Para a IG ≥ 28 semanas, ocorreu estabilidade de 2008 a 2014, com retomada da redução de 2014 a 2021.

Durante a pandemia de covid-19, o número de natimortos apresentou variações específicas por estratos de IG analisados. Na IG ≥ 20 semanas, houve uma diminuição de 0,38% em 2020, seguida de um aumento de 1,15% em 2021, resultando em um aumento geral de 0,76% no biênio. Por outro lado, na IG ≥ 22 semanas, observou-se uma redução de 1% em 2020 e um aumento de 0,9% em 2021, levando a uma redução de 0,09% no biênio. Quanto à IG ≥ 28 semanas, os natimortos aumentaram 0,75% em 2020 e reduziram 0,94% em 2021, culminando em uma redução de 0,19% no biênio.

As TMF nos estratos de IG ≥ 20, ≥ 22 e ≥ 28 semanas apresentaram uma tendência decrescente de longo prazo no período analisado. As VPMA foram de -1,00, -1,13 e -1,89, respectivamente, sendo estatisticamente significativas. Notavelmente, a VPMA da série temporal das TMF na IG ≥ 20 semanas foi 12% inferior à verificada na IG ≥ 22 semanas e 47% menor do que a observada na IG ≥ 28 semanas. As TFM reduziram 20%, 25% e 41%, respectivamente, nos estratos de IG analisados, entre 1996 e 2021.

Antes da pandemia de covid-19 (1996–2019), a VPMA negativa da série temporal das TMF foi 1,06, 1,26 e 1,05 vezes maior, respectivamente, nos estratos de IG analisados, em comparação com a VPMA negativa verificada no período incluindo a pandemia (1996–2021) nos mesmos estratos. Entretanto, a tendência decrescente de longo prazo na mortalidade fetal não foi interrompida. Na análise de tendências interrompidas, observaram-se períodos específicos de aumento, diminuição ou estabilização nas séries temporais das TMF, nos estratos de IG analisados.

### Determinantes Sociodemográficos e Obstétricos

Os dados revelam disparidades e desigualdades da mortalidade fetal influenciadas por características demográficas e obstétricas no Brasil, entre 1996 e 2021. A Tabela 2 apresenta as taxas globais de mortalidade fetal e a análise de tendência de longo prazo nas séries temporais das TMF anuais nos estratos de IG ≥ 22 e ≥ 28 semanas por características sociodemográficas, para os períodos anterior e incluindo a pandemia de covid-19 no Brasil, entre 1996 e 2021. Observou-se que, apesar das reduções verificadas, persistiram desigualdades na mortalidade fetal associadas ao sexo do óbito fetal, à idade materna e à escolaridade materna.

**Tabela 2:** Taxa global de mortalidade fetal e análise de tendências de longo prazo nas séries temporais das taxas de mortalidade fetal nos estratos de idade gestacional ≥ 22 e ≥ 28 semanas por características sociodemográficas para antes e incluindo a pandemia de covid-19. Brasil, 1996–2021.

Variáveis	Mortalidade fetal							
	Idade gestacional ≥ 22 semanas				Idade gestacional ≥ 28 semanas			
	1996–2019^**^		1996–2021^***^		1996–2019^**^		1996–2021^***^	
	TMF^****^	VPMA^*^	TMF^****^	VPMA^*^	TMF^****^	VPMA^*^	TMF^*****^	VPMA^*^
Sexo do óbito								
Masculino	11,3	-1,19^*^	11,3	-1,12^*^	9,1	-2,07^*^	9	-2,00^*^
Feminino	10,1	-1,01^*^	10,1	-0,93^*^	8,2	-1,61^*^	8,1	-1,53^*^
Idade materna (anos)								
10–14	13,6	-0,36^*^	13,7	-0,22^*^	9,2	-1,43^*^	9,2	-1,26^*^
15–19 anos	10,2	-0,55^*^	10,3	-0,40^*^	7,8	-1,41^*^	7,8	-1,26^*^
20–24 anos	9,6	-0,64^*^	9,5	-0,59^*^	7,6	-1,58^*^	7,6	-1,56^*^
25–29 anos	9,7	-1,26^*^	9,6	-1,39^*^	7,9	-1,88^*^	7,8	-1,80^*^
30–34 anos	11	-2,17^*^	10,8	-1,72^*^	9	-2,85^*^	8,8	-2,46^*^
35–39 anos	14,8	-2,86^*^	14,4	-2,51^*^	12,3	-3,36^*^	11,9	-3,33^*^
40–44 anos	23	-2,65^*^	22,3	-2,77^*^	19,7	-3,07^*^	18,9	-3,07^*^
45–49 anos	34,1	-1,64^*^	33,1	-1,77^*^	29,8	-2,15^*^	28,8	-2,27^*^
Escolaridade materna (em anos)								
Nenhuma	31,7	9,45^*^	32,4	9,21^*^	24,6	8,30^*^	25,1	8,03^*^
1–3 anos	13,4	7,35^*^	13,6	7,16^*^	10,2	6,62^*^	10,4	6,40^*^
4–7 anos	12,6	-1,94^*^	12,7	-1,54^*^	9,2	-2,96^*^	9,4	-2,48^*^
8–11 anos	9,2	-2,89^*^	9,1	-2,65^*^	8,4	-4,17^*^	8,2	-3,93^*^
≥ 12 anos	7,2	-0,77^*^	7,1	-0,69^*^	4,9	1,60^*^	4,8	-1,07^*^

TMF: taxa de mortalidade fetal.

Fonte: baseado em dados do Ministério da Saúde e Sistema de Informações sobre Mortalidade, 2022.

^*^Indica que a variação percentual media anual (VPMA) e significativamente diferente de zero no nível alfa = 0,05, quando o resultado for positivo, tendencia crescente, se negativo, tendencia decrescente. Quando a VPMA não for significativamente diferente de zero, a tendencia e estacionaria. ^**^Período anterior a pandemia de covid-19. ^***^Período que incluiu a pandemia de covid-19. ^****^Taxa global de mortalidade fetal por 1.000 nascimentos totais (nascidos vivos + natimortos) do total do período 1996–2019 e 1996–2021.

O padrão das taxas globais de mortalidade fetal para IG ≥ 22 e IG ≥ 28 semanas é semelhante, com TMF globais menores na IG ≥ 28 semanas, no total do período de 1996 a 2021. O risco global estimado de um feto nascer sem qualquer sinal de vida por 1.000 nascimentos totais (nascidos vivos + natimortos), no período analisado, foi maior para o sexo masculino do que para o feminino, tanto no período anterior quanto no período incluindo a pandemia de covid-19.

Nos extremos de idades maternas, ou seja, em gestantes com idade avançada, especialmente entre 45 e 49 anos, e em adolescentes, principalmente entre 10 e 14 anos, o risco estimado de mortalidade fetal foi mais elevado. As TMF globais foram 3,5 vezes maiores na idade materna mais avançada (45–49 anos), em comparação com as menores taxas observadas na faixa etária de 20 a 24 anos, quando considerada a IG ≥ 22 semanas. Já na IG ≥ 28 semanas, as TMF globais na idade materna mais avançada (45–49 anos) foram 3,8 vezes maiores do que as taxas mínimas registradas na faixa etária de 20 a 24 anos.

Ademais, as gestantes sem escolaridade apresentaram maior risco de mortalidade fetal em comparação com aquelas com algum nível de instrução, e observou-se uma tendência de TMF progressivamente menores à medida que o nível de escolaridade aumentava. As TMF globais foram particularmente elevadas entre mulheres sem instrução formal, sendo 4,5 vezes maiores na IG ≥ 22 semanas e 5,2 vezes maiores na IG ≥ 28 semanas, quando comparadas às TMF mínimas registradas para mulheres com 12 anos ou mais de escolaridade.

Em relação ao padrão de tendência, embora as TMF tenham sido mais elevadas no sexo masculino, observou-se uma redução mais acentuada dessas taxas em comparação ao sexo feminino. Verificou-se uma tendência decrescente significativa de longo prazo das TMF para todos os grupos etários de mães e níveis de escolaridade materna, com exceção das mulheres sem escolaridade e com um a três anos de estudo, as quais apresentaram tendência crescente. As maiores reduções nas TMF global em relação à idade materna ocorreram nas faixas etárias entre 25 e 44 anos.

Ao comparar os períodos antes (1996–2019) e incluindo a pandemia de covid-19, observou-se que as TMF globais permanecem praticamente inalteradas para ambos os sexos do óbito fetal, faixas etárias maternas e níveis de escolaridade materna. No entanto, a VPMA negativa, que indica uma tendência decrescente das TMF, foi menor no período incluindo a pandemia para a maioria dos grupos analisados, sugerindo uma desaceleração no declínio da mortalidade fetal. Esse padrão de desaceleração no declínio se mostrou semelhante para os estratos de IG analisados.

A desaceleração na tendência decrescente de longo prazo das TMF do total do período pode ser explicada pelos aumentos significativos nessas taxas observados em 2020, principalmente nos estratos sociodemográficos mais vulneráveis. Esses aumentos foram sucedidos por acréscimos de menor magnitude em 2021, acompanhados de algumas reduções pontuais.

A Tabela 3 apresenta as taxas globais de mortalidade fetal e a análise de tendências de longo prazo nas séries temporais das TMF anuais, considerando os estratos de IG (≥ 22 e ≥ 28 semanas), por características obstétricas, para os períodos antes e incluindo a pandemia de covid-19 no Brasil, entre 1996 e 2021. Observou-se desigualdade no risco de mortalidade fetal associada ao local de ocorrência do óbito fetal, à duração e tipo de gravidez, ao tipo de parto e ao peso ao nascer no país.

**Tabela 3:** Taxa global de mortalidade fetal e análise de tendências de longo prazo nas séries temporais das taxas de mortalidade fetal nos estratos de idade gestacional ≥ 22 e ≥ 28 semanas segundo características obstétricas para antes e incluindo a pandemia de covid-19. Brasil, 1996–2021.

Variáveis	Mortalidade fetal							
	Idade gestacional ≥ 22 semanas				Idade gestacional ≥ 28 semanas			
	1996–2019^**^		1996–2021^***^		1996–2019^**^		1996–2021^***^	
	TMF^****^	VPMA^*^	TMF^****^	VPMA^*^	TMF^****^	VPMA^*^	TMF^****^	VPMA^*^
Local de ocorrência								
Hospital	10,3	-1,12^*^	10,3	-1,05^*^	8,3	-2,10^*^	8,2	-2,03^*^
Outro estabelecimento de saúde	11,9	1,36^*^	12,3	1,83^*^	10	0,57^*^	10,3	1,05
Domicílio	34,8	0,42^*^	35,4	0,51^*^	29	-0,3	29,5	-0,19^*^
Outro	119,5	-3,57^*^	109,2	-4,38^*^	105	-3,97^*^	95,6	-4,76^*^
Duração da gravidez (semanas)								
22–27	381,2	-0,01	378	0,08	-	-	-	-
28–31	207,5	-4,07^*^	203,2	-3,77^*^	218	-3,97^*^	213,1	-3,69^*^
32–36	41,5	-3,37^*^	40,2	-3,11^*^	44	-4,30^*^	42,6	-3,98^*^
37–41	3,2	-0,86^*^	3,2	-0,94^*^	3,4	-1,42^*^	3,4	-1,37^*^
≥ 42	7,5	-6,30^*^	7,1	-5,96^*^	8	-6,47^*^	7,6	-6,11^*^
Tipo de gravidez								
Única	10,4	-1,10^*^	10,3	-1,03^*^	8,4	-1,90^*^	8,3	-1,81^*^
Dupla	28,5	-1,67^*^	28,1	-1,61^*^	21	-3,09^*^	20,5	-3,06^*^
Tripla e mais	41,6	-2,86^*^	41	-2,57^*^	31,1	-4,59^*^	30,5	-4,26^*^
Tipo de parto								
Vaginal	14,4	-0,29^*^	14,4	0,02^*^	10,8	-1,43^*^	10,8	-1,03^*^
Cesáreo	6,7	-2,08^*^	6,6	-1,70^*^	6,2	-2,42^*^	6,1	-2,00^*^
Peso ao nascer (g)								
< 500	220,4	-0,12	230,5	0,25	92,3	-4,01^*^	91,2	-3,75^*^
500–999	354,7	-0,53^*^	352,7	-0,49^*^	170,7	-2,47^*^	168,7	-2,30^*^
1.000–1.499	191	-1,24^*^	189,3	-0,92^*^	160,2	-1,53^*^	159,1	-1,34^*^
1.500–2.499	39,4	-1,25^*^	39,1	-1,21^*^	39,1	-1,38^*^	38,8	-1,32^*^
2.500–2.999	5,6	-1,43^*^	5,6	-1,37^*^	5,7	-1,57^*^	5,7	-1,51^*^
3.000–3.999	2,5	-1,99^*^	2,5	-1,99^*^	2,6	-2,17^*^	2,5	-2,17^*^
≥ 4.000	6,4	-2,09^*^	6,3	-2,14^*^	6,5	-2,23^*^	6,4	-2,30^*^

TMF: taxa de mortalidade fetal; g: gramas

Fonte: baseado em dados do Ministério da Saúde e Sistema de Informações sobre Mortalidade, 2022.

^*^Indica que a variação percentual média anual (VPMA) é significativamente diferente de zero no nível alfa = 0,05, se resultado positivo, tendência crescente, se negativo, decrescente. Quando a VPMA positiva ou negativa não for significativamente diferente de zero, estabilidade. ^**^Período anterior à pandemia de covid-19. ^***^Período que incluiu a pandemia de covid-19. ^****^Taxa global de mortalidade fetal por 1.000 nascimentos totais (nascidos vivos + natimortos) do total dos períodos: 1996–2019 e 1996–2021.

Semelhante ao observado para as características sociodemográficas das mortes fetais (Tabela 2), por variáveis obstétricas, as maiores taxas globais de mortalidade fetal para o período analisado, considerados ambos os estratos de IG analisados na Tabela 3, ocorreram para a IG ≥ 22 semanas, quando comparadas com a IG ≥ 28 semanas. Para gestações ≥ 20 e < 22 semanas, embora não incluídas na Tabela 3, as TMF globais foram ainda maiores em comparação com a IG ≥ 22 e ≥ 28 semanas.

Nos grupos de variáveis obstétricas, as TMF globais mais elevadas para o período de 1996 a 2021 ocorreram em partos domiciliares e outras localizações, como aldeias indígenas e vias públicas. Além disso, a TMF global foi mais acentuada em gestações com duração inferior a 37 semanas, sendo ainda mais elevada naquelas inferiores a 31 semanas, bem como em gestações gemelares e gravidez tripla ou superior. Também foram observadas TMF globais mais elevadas em partos vaginais e baixo e em natimortos com peso ao nascer (< 2.500 g), com maior concentração na faixa de peso entre 500 e 999 g.

Em relação ao local de ocorrência do óbito fetal, a TMF global mais elevada em outros locais, como vias públicas e aldeias indígenas, foi 11,6 vezes maior do que a menor TFM global observada em hospitais. A TMF global mais alta em gestações de 22 a 27 semanas foi 119 vezes superior à mais baixa, observada nas gestações de 37 a 41 semanas. Nas gestações múltiplas, a TMF global foi aproximadamente quatro vezes maior em comparação às gestações únicas. Ademais, a TMF global foi duas vezes maior nos partos vaginais em relação aos partos cesáreos. Quanto ao peso ao nascer, a TMF global mais elevada na faixa de 500 a 999 g foi 142 vezes superior à observada na faixa de 3.000 a 3.999 g.

Tendências decrescentes de longo prazo significativas foram observadas na maioria das categorias dos grupos de características obstétricas analisados. As TMF anuais apresentaram as maiores reduções em outros locais e hospitais, na gestação com 42 semanas ou mais, e entre 28 e 31 semanas, na gravidez gemelar múltipla, no parto cesáreo e no peso ao nascer de 4.000 g ou mais. Especificamente, no estrato de IG ≥ 28 semanas, a TMF anual diminuiu drasticamente para o peso ao nascer < 500 g. As exceções de aumento das TMF anuais ocorreram para outros estabelecimentos de saúde e partos domiciliares (para IG ≥ 22 semanas), e tendência estacionária foi observada para o peso ao nascer < 500 g.

Ao comparar os períodos antes (1996–2019) e incluindo a pandemia (1996–2021), observou-se que tanto as TMF globais quanto as tendências nas séries temporais das TMF anuais foram bastante semelhantes para a maioria das categorias analisadas. No entanto, a pandemia de covid-19 teve um impacto significativo, causando uma desaceleração nas tendências decrescente de longo prazo e uma aceleração nas tendências crescentes, como observado em outro estabelecimento de saúde.

### Fatores Perinatais Responsáveis por Complicações Fetais

As complicações fetais responsáveis por causar a maioria das mortes fetais foram desencadeadas por fatores perinatais e malformações congênitas, cujo impacto poderia ser mitigado por melhorias na assistência pré-natal e durante o parto, tanto na atenção primária quanto na atenção hospitalar. A Tabela 4 apresenta o número total de natimortos, a proporção correspondente e a análise de tendência de longo prazo nas séries temporais anuais de natimortos nos estratos de IG ≥ 22 e ≥ 28 semanas por capítulo e lista de causas de morte da CID-10, no Brasil, para o período de 1996 a 2021.

**Tabela 4:** Número total de mortes fetais, proporção e a análise de tendência nas séries temporais de óbitos fetais nos estratos de idade gestacional ≥ 22 e ≥ 28 semanas para antes e incluindo a pandemia de covid-19 por capítulo e lista de causas de mortes da CID-10. Brasil, 1996–2021.

Capítulo e lista de causas de morte CID-10	Mortalidade fetal									
	Idade gestacional ≥ 22 semanas					Idade gestacional ≥ 28 semanas				
	1996–2019^**^		1996–2021^***^			1996–2019^**^		1996–2021^***^		
	n^****^	VPMA^*^	n^****^	(ρ)^*****^	VPMA^*^	n^****^	VPMA^*^	n^****^	(ρ)^*****^	VPMA^*^
Algumas doenças infecciosas e parasitárias	4.394	11,46^*^	5.220	-0,65	10,56^*^	3.397	13,41^*^	4.064	-0,65	12,43^*^
Neoplasias	2	-	2	-0,0003	-	2	-	2	-0,0003	-
Algumas afecções originadas no período perinatal	699.815	0,23	749.265	-94	0,13	554.368	-0,16	591.847	-94,05	-0,28
Feto e recém-nascido afetados por fatores maternos e por complicações da gravidez, do trabalho de parto e do parto	275.748	1,77^*^	299.793	-37,61	1,70^*^	216.894	1,43^*^	234.839	-37,32	1,36^*^
Transtornos relacionados com a duração da gestação e com o crescimento fetal	10.381	-6,03^*^	11.201	-1,41	-3,65^*^	7.022	-6,17^*^	7.527	-1,2	-5,80^*^
Traumatismo ocorrido durante o nascimento	563	2,35	583	-0,07	1,8	490	2,29	509	-0,08	1,77
Hipóxia intrauterina e asfixia ao nascer	187.255	-0,83^*^	196.976	-24,71	-1,72^*^	148.838	-1,37^*^	156.230	-24,83	-1,90^*^
Desconforto (angústia) respiratório(a) do recém-nascido	119	-10,42^*^	119	-0,01	-10,42^*^	92	-9,57^*^	92	-0,01	-9,57^*^
Pneumonia congênita	41	-15,05^*^	41	-0,01	-14,02^*^	40	-15,13^*^	40	-0,01	-14,10^*^
Outras afecções respiratórias do recém-nascido	1.103	-13,29^*^	1.124	-0,14	-12,44^*^	979	-13,47^*^	996	-0,16	-12,63^*^
Septicemia bacteriana do recém-nascido	42	-7,02^*^	42	-0,01	-7,02^*^	36	-6,85^*^	36	-0,01	-6,85^*^
Transtornos hemorrágicos e hematológicos do feto	2.911	-1,01^*^	3.184	-0,4	-0,24	2.335	-1,96^*^	2.514	-0,4	-1,96^*^
Restante das afecções perinatais	221.652	-0,67^*^	236.202	-29,63	-0,41	177.642	-0,85^*^	189.064	-30,05	-0,5
Malformações congênitas, deformidades e anomalias	38.067	2,48^*^	41.593	-5,22	2,40^*^	30.007	1,59^*^	32.424	-5,15	1,50^*^
Hidrocefalia e espinha bífida congênitas	2.090	-2,26^*^	2.172	-0,27	-3,34^*^	1.700	-3,52^*^	1.755	-0,28	-4,08^*^
Outras malformações congênitas do sistema nervoso	9.730	0,6	10.351	-1,3	0,45	7.938	-0,24	8.338	-1,33	-0,53
Malformações congênitas do coração	3.288	4,11^*^	3.695	-0,46	4,11^*^	2.769	3,41^*^	3.084	-0,49	3,41
Outras malformações congênitas do aparelho circulatório	362	8,87^*^	391	-0,05	4,85^*^	287	5,78^*^	308	-0,05	1,69
Síndrome de Down e outras anomalias cromossômicas	2.500	10,49^*^	2.985	-0,37	10,26^*^	1.900	9,92^*^	2.243	-0,36	9,65^*^
Outras malformações congênitas	20.097	2,45^*^	21.999	-2,76	2,37^*^	15.413	1,45^*^	16.696	-2,65	1,21^*^
Sintomas, sinais e achados anormais de exames clínicos	972	-22,85^*^	972	-0,12	-21,25^*^	917	-22,68^*^	917	-0,15	-21,09^*^
Síndrome da morte súbita na infância	10	-2,25	10	-0,001	-2,05	9	-2,33	9	-0,001	-2,11
Outros sintomas, sinais e achados anormais de exames	962	-22,81^*^	962	-0,12	-21,17^*^	908	-22,62^*^	908	-0,14	-21,00^*^
Todas as outras doenças	1	-	1	-0,0001	-	1	-	1	-0,0002	-
Causas externas de morbidade e mortalidade	8	-9,11^*^	8	-0,001	-8,45^*^	8	-9,11^*^	8	-0,001	-8,45^*^

^*^Indica que a variação percentual média anual (VPMA) é significativamente diferente de zero no nível alfa = 0,05, se resultado positivo, tendência crescente, se negativo, decrescente. Quando a VPMA positiva ou negativa não for significativamente diferente de zero, estabilidade. ^**^Período anterior à pandemia de covid-19. ^***^Período que incluiu a pandemia de covid-19. ^****^População total de mortes fetais por capítulo e lista de causas de mortes da CID-10 nos estratos de idade gestacional ≥ 22 e ≥ 28 semanas. ^*****^Proporção.

Fonte: baseado em dados do Ministério da Saúde e Sistema de Informação sobre Mortalidade, 2022.

As principais causas de mortes fetais nos estratos de IG analisados foram as afecções originadas no período perinatal (94%) – Capítulo XVI da CID-10, destacando-se “Feto e recém-nascido afetados por fatores maternos e por complicações da gravidez, do trabalho de parto e do parto” (38%) e “Hipóxia intrauterina e asfixia ao nascer” (25%). Malformações congênitas, deformidades e anomalias cromossômicas (Capítulo XVII da CID-10) também representaram uma causa relevante (5%), com destaque para “Outras malformações congênitas do sistema nervoso” (1,3%) e “Síndrome de Down e outras anomalias cromossômicas” (0,37%).

Observou-se uma tendência crescente de longo prazo nas mortes fetais relacionadas aos agrupamentos de causas por capítulos da CID-10, com exceção do Capítulo XVIII (“Sintomas, sinais e achados anormais de exames clínicos e de laboratório, não classificados em outra parte”), que apresentou tendência decrescente, e do Capítulo XVI, que se manteve estável. Durante o período da pandemia de covid-19 (2020–2021), houve desaceleração na tendência crescente e na decrescente relativas aos agrupamentos por capítulo de causas de mortes fetais.

A tendência crescente a longo prazo no número de mortes fetais, desagregados pela lista de causas de morte, concentrou-se especificamente nas afecções perinatais, particularmente no grupo “feto e recém-nascido afetados por fatores maternos e por complicações da gravidez, do trabalho de parto e do parto” (VPMA de 1,70 na IG ≥ 22 semanas e 1,36 na IG ≥ 28 semanas), e nas malformações congênitas, com maior crescimento nas mortes fetais por “Síndrome de Down e outras anomalias cromossômicas” (VPMA de 10,26 na IG ≥ 22 semanas e 9,65 na IG ≥ 28 semanas). Essa tendência de aumento e de redução foi atenuada durante o período da pandemia de covid-19.

Uma parcela significativa das mortes fetais (67%) no Brasil, entre 1996 e 2021, era potencialmente evitável, sendo 39% reduzíveis por meio de assistência adequada à gestante durante a gestação e o parto na Atenção Primária à Saúde, e 28% evitáveis com assistência hospitalar qualificada durante o parto. Ações de imunoprevenção poderiam ter reduzido 0,65% das mortes fetais classificados na Atenção Primária à Saúde. Esses dados ressaltam a importância de investimentos e melhorias na assistência pré-natal, no parto e em medidas de prevenção para reduzir a mortalidade fetal no país durante o período analisado.

### Diferenças Regionais

Os dados analisados revelam diferenças regionais na distribuição dos natimortos e TMF no Brasil entre 1996 e 2021. As regiões Sudeste e Nordeste concentraram os maiores números absolutos de natimortos no país, considerando as IG ≥ 20 e ≥ 22 semanas, respectivamente. A região Sudeste apresentou média anual de 13.483 (± 3.014 desvio padrão) e 12.581 (± 2.905 DP) natimortos, enquanto o Nordeste registrou média anual de 10.996 (± 924 DP) e 10.416 (± 995 DP) natimortos.

Embora com menores números absolutos, as regiões Norte e Nordeste apresentaram as maiores taxas brutas médias relativas de natimortos por 1.000 nascimentos totais nos estratos de IG analisados. A região Norte teve média anual de 11,7 (± 11,7 DP) e 11,0 (± 0,9 DP) natimortos por 1.000 nascimentos totais, enquanto o Nordeste registrou média anual de 12,6 (± 0,6 DP) e 12,0 (± 0,6 DP) natimortos por 1.000 nascimentos totais, para IG ≥ 20 e ≥ 22 semanas, respectivamente.

A região Centro-Oeste apresentou o menor número absoluto de natimortos, com média anual de 2.331 (± 156 desvio-padrão) e 2.207 (± 180 DP), considerando IG ≥ 20 e ≥ 22 semanas, respectivamente. No entanto, a região Sul exibiu a menor taxa bruta de mortalidade fetal, com média anual de 9,1 (± 1,1 DP) e 8,7 (± 1,3 DP) óbitos por 1.000 nascimentos totais, respectivamente, para os mesmos estratos de IG analisados.

As regiões Norte e Sul apresentaram número absoluto mediano de natimortos, com 3.562 (± 132 de desvio padrão) e 3.343 (± 159 DP), e 3.738 (± 768 DP) e 3.573 (± 811 DP), respectivamente, considerando IG ≥ 20 e ≥ 22 semanas. No entanto, foram as regiões Sudeste e Centro-Oeste que exibiram TMF bruta mediana, com média anual de 11,3 (± 1,9 DP) e 10,6 (± 1,9 DP) natimortos por 1.000 nascimentos totais na região Sudeste, e 9,9 (± 0,6 DP) e 9,4 (± 0,7 DP) natimortos por 1.000 nascimentos totais na região Centro-Oeste, nos mesmos estratos de IG analisados. O maior número absoluto de natimortos na região Sudeste pode ser atribuído à maior população (42,28%) e maior número de nascimentos (39,43%) nessa região.

As TMF médias anuais padronizadas, considerando IG ≥ 22 semanas, ajustadas pelos parâmetros nacionais do Brasil, das regiões Norte (10,4 óbitos por 1.000 nascimentos totais; desvio padrão ± 0,9), Nordeste (10,6/1.000; DP ±1,0) e Sudeste (10,5 ± 1,9 DP) ficaram semelhantes e abaixo da TMF média nacional (10,7/1.000; DP ±1,0) entre 1996 e 2021. Contudo, a TMF média anual padronizada da região Nordeste ficou superior às observadas nas regiões Sudeste, Norte, Sul (8,6 ± 1,2 DP) e Centro-Oeste (9,7 ± 0,9 DP) no período analisado. De 1996 a 2000 e em 2002, as TMF padronizadas da região Sudeste foram maiores que as TMF nacionais.

Todas as regiões apresentaram uma tendência geral de queda no número de mortes fetais ao longo do período analisado (1996–2021), considerando a IG ≥ 20 e ≥ 22 semanas, com algumas flutuações pontuais. A região Sudeste registrou a queda mais acentuada, com redução de 48% e 51%, respectivamente.

As regiões Norte e Nordeste também exibiram reduções significativas no número de mortes fetais, embora com valores absolutos menores. A região Norte apresentou redução de 12% e 4%, enquanto a região Nordeste apresentou queda de 3% e 5%, considerando as IG ≥ 20 e ≥ 22 semanas, respectivamente. A região Sul apresentou a segunda maior redução, com 41% e 46%, e a região Centro-Oeste apresentou redução mediana de 3% e 5,6%, respectivamente.

A Tabela 5 apresenta a análise de tendência de longo prazo e tendência interrompida nas séries temporais das taxas brutas de mortalidade fetal anuais para os anos anteriores e incluindo os anos da pandemia de covid-19, segundo as Grandes Regiões no Brasil, entre 1996 e 2021. A maioria das regiões do país apresentou uma tendência decrescente de longo prazo nas TMF brutas ao longo do período analisado. No entanto, a região Centro-Oeste permaneceu estacionária nesse período, não apresentando variações significativas na TMF bruta.

**Tabela 5:** Análise de tendência de longo prazo e tendência interrompida nas séries temporais das taxas brutas de mortalidade fetal para antes e incluindo a pandemia de covid-19 segundo a Grande Região. Brasil, 1996–2021.

Variáveis	Segmento	VPA	IC95%	VPMA	IC95%	Interpretação
Norte	1996–2000	-3,45^*^	(-7,31 a -1,45)			Decrescente
	2000–2014	-1,03	(-1,50 a 0,06)			Estacionária
	2014–2021	1,23^*^	(0,12 a 4,00)			Crescente
	1996–2019			-0,97^*^	(-1,24 a -0,70)	Decrescente
	1996–2021			-0,79^*^	(-1,06 a -0,51)	Decrescente
Nordeste	1996–1999	-3,55^*^	(-8,14 a -1,24)			Decrescente
	1999–2002	6,28^*^	(3,37 a 7,78)			Crescente
	2002–2021	-0,71^*^	(-0,91 a -0,57)			Decrescente
	1996–2019			-0,21	(-0,39 a 0,03)	Estacionária
	1996–2021			-0,25^*^	(-0,42 a -0,03)	Decrescente
Sudeste	1996–1998	-8,83^*^	(-10,84 a -3,92)			Decrescente
	1998–2008	-3,44^*^	(-4,00 a -0,52)			Decrescente
	2008–2021	-0,2	(-0,62 a 0,42)			Estacionária
	1996–2019			-2,39^*^	(-2,56 a -2,04)	Decrescente
	1996–2021			-2,22^*^	(-2,39 a -1,89)	Decrescente
Sul	1996–1999	1,82	(-0,27 a 5,50)			Estacionária
	1999–2008	-3,06^*^	(-4,12 a -2,62)			Decrescente
	2008–2021	-1,14^*^	(-1,41 a -0,80)			Decrescente
	1996–2019			-1,52^*^	(-1,68 a -1,32)	Decrescente
	1996–2021			-1,49^*^	(-1,64 a -1,30)	Decrescente
Centro-Oeste	1996–2001	1,60^*^	(0,22 a 4,88)			Crescente
	2001–2005	-3,84^*^	(-5,93 a -1,67)			Decrescente
	2005–2019	-0,46	(-1,23 a 0,13)			Estacionária
	2019–2021	6,03^*^	(0,07 a 8,96)			Crescente
	1996–2019			-0,61^*^	(-0,79 a -0,23)	Decrescente
	1996–2021			-0,10	(-0,47 a 0,20)	Estacionária

IC95%: intervalo de confiança de 95%.

Fonte: baseado em dados do Ministério da Saúde e Sistema de Informação sobre Mortalidade, 2022.

*Indica que a variação percentual anual (VPA) e a variação percentual anual média (VPMA) são significativamente diferentes de zero no nível alfa = 0,05. A interpretação de tendência interrompida é dada pela VPA, e de tendência de longo prazo é pela VPMA.

A menor redução nas TMF bruta, considerando IG ≥ 22 semanas, ocorreu na região Nordeste (2%), com VPMA de -0,25, indicando uma tendência decrescente de longo prazo, embora tenha permanecido estacionária entre 1996 e 2019. Em seguida, a região Norte apresentou tendência decrescente, reduzindo 19% na TMF bruta, com VPMA de -0,42; e o Centro-Oeste reduziu 1%, com VPMA de -0,79. As regiões que apresentaram as maiores reduções nas TMF brutas foram a Sudeste (41%), com VPMA de -2,04, e a Sul (29%), com VPMA de -1,14.

Ao considerar as TMF padronizadas para a IG ≥ 22 semanas, observaram-se reduções mais expressivas do que as observadas nas TMF brutas. Na região Norte, houve uma redução de 23% (VPMA -1,08; IC95% -1,25 a -0,85), na região Nordeste, uma redução de 25% (VPMA -1,23; IC95% -1,41 a -0,99), e na região Centro-Oeste, uma redução de 6,7% (VPMA -0,34; IC95% -0,76 a -0,05), todas estatisticamente significativas. Por outro lado, a região Sudeste manteve uma redução semelhante (41%), enquanto a região Sul revelou redução de 26% (VPMA -1,38; IC95% -1,54 a -1,13), menor do que a redução observada na TMF bruta.

Observou-se, ao longo do período analisado, que ocorreram intervalos nos quais as TMF não apresentaram alterações significativas nas Grandes Regiões. Em outras palavras, identificaram-se segmentos temporais de estabilização dessas taxas. Na região Sudeste, as TMF se estabilizaram no período recente, de 2008 a 2021. Na região Sul, as TMF exibiram estabilidade entre os anos de 1996 e 1999. A região Norte revelou estabilidade nas TMF no intervalo de 2000 a 2014, enquanto o Centro-Oeste apresentou taxas estáveis entre 2005 e 2019.

As regiões Sudeste e Sul mantiveram tendências decrescentes semelhantes das TMF antes e incluindo a pandemia de covid-19, com uma desaceleração na redução nos anos pandêmicos. Por outro lado, as demais regiões apresentaram comportamentos distintos. A região Norte exibiu uma desaceleração na redução da TMF, enquanto o Nordeste transitou de uma tendência estacionária para decrescente. Já o Centro-Oeste reverteu de uma tendência decrescente para estacionária quando foram incluídos os dados referentes ao período pandêmico. Esses dados sugerem que a covid-19 teve impactos diferenciados nas tendências regionais de mortalidade fetal no Brasil.

## DISCUSSÃO

### Tendencias Nacionais

O Brasil registrou progresso substancial na sobrevivência fetal entre 1996 e 2021, no entanto, os resultados evidenciam que a mortalidade fetal constituiu grave problema de saúde pública no país, com taxas consideradas preocupantes e lacunas de informações^29,30^. As taxas médias anuais de mortalidade fetal foram 11,3, 10,7 e 8,5 natimortos por 1.000 nascimentos totais, para IG ≥ 20, ≥ 22 e ≥ 28 semanas, respectivamente. Essas taxas indicam elevado risco de morte fetal no país, superior ao observado em nações da América Latina e Caribe^9^ e países desenvolvidos, demandando intervenções e medidas para reduzir mortes fetais evitáveis^12,31^.

O avanço foi acompanhado por uma ocorrência expressiva de mortes fetais com IG igual ou superior a 20 semanas. Constatou-se, ainda, redução anual da mortalidade fetal inferior à da mortalidade infantil. Adicionalmente, a tendência decrescente de longo prazo da mortalidade fetal foi impactada pela pandemia de covid-19 em 2020 e 2021^32,33^, com uma desaceleração no ritmo de redução das TMF, porém não foi interrompida. Antes da pandemia (1996–2019), a redução na TMF foi superior à verificada no período incluindo os anos de pandemia (1996–2021).

Nos últimos anos, desde 1996 até 2021, o Brasil registrou expressiva ocorrência de natimortos com IG ≥ 20 semanas, totalizando 886.878 casos, o equivalente a aproximados 70 natimortos diários. Desse montante, 835.153 (94%) ocorreram com IG ≥ 22 semanas, e 665.287 (75%) na IG ≥ 28 semanas, considerados fetos viáveis^34^. Anualmente, foram registrados aproximadamente 25 mil natimortos com IG ≥ 20 semanas, 32 mil com IG ≥ 22 semanas ou 34 mil com IG ≥ 28 semanas. Esses dados indicam a importância da necessidade de intervenções para reduzir a natimortalidade.

Na América Latina e Caribe, entre 2000 e 2021, a parcela de natimortos brasileira com IG ≥ 28 semanas aumentou 17,2%: saiu de 23% de participação em 2000 e passou para 27% em 2021, mostrando piora no indicador em relação à média regional e necessidade de maior atenção do país. No cenário global, o Brasil aumentou em 4% sua participação nessa mortalidade, de 1,03% de 2000 chegou a 1,07 em 2021, na IG ≥ 28 semanas, um quadro preocupante de mortalidade fetal^9^.

Ao comparar as TMF no Brasil, considerando a IG ≥ 28 semanas, que foram 12,7 óbitos por 1.000 nascimentos totais em 2000 e 7,5 em 2021, com as da América Latina e Caribe, 11,2/1.000 em 2000 e 7,7 em 2021, observou-se que o país apresentou taxa inferior em 2021. Em âmbito mundial, cujas taxas na IG ≥ 28 semanas foram 21,3/1.000 em 2000 e 13,9 em 2021, o país exibiu taxas inferiores no período analisado^9^.

O alto o percentual de 1,14% de perdas fetais com IG ≥ 20 semanas em relação aos nascimentos totais é um indicador preocupante que poderia ser mitigado em 67% com intervenções efetivas^35,36^ e políticas públicas adequadas, pois reflete falhas nos sistemas de saúde materno-infantil^35,37^. Mas preocupante é a ocorrência de mortes fetais em fetos viáveis, com IG ≥ 22 semanas, que corresponderem a aproximados 57% das mortes perinatais a partir da IG ≥ 22 semanas e idade de 0 a 6 dias de vida. Para mortes perinatais a partir da IG ≥ 28 semanas, 51% foram mortes fetais, em fetos ainda mais viáveis^9^.

A presença significativa de mortes fetais em fetos viáveis, somada à possível subnotificação, indica a necessidade de intervenções nas diferentes fases do ciclo gestacional para aumentar as chances de sobrevivência fetal. O monitoramento das mortes fetais precoces, principalmente das evitáveis, que requerem maior atenção e investimento, é fundamental para o processo de prevenção e controle da mortalidade fetal, para reduzi-la^38^.

Os progressos feitos na redução da TMF no Brasil, no período analisado, é um indicador importante de melhoria na saúde materno-infantil, representando avanços na sobrevivência fetal e perinatal no país^39^. Essas taxas apresentaram tendência decrescente de longo prazo, com taxas anuais de redução de 1,0%, 1,1% e 1,89%, para IG ≥ 20, ≥ 22 e ≥ 28 semanas. No entanto, verificou-se que as taxas de sobrevivência fetal variam de acordo com a IG e determinantes^42^.

Considerando a IG ≥ 28 semanas, a taxa anual de redução da TMF no Brasil foi de 0,85% (IC95% -1,0 a -0,69) entre 1996 e 2021. Essa taxa anual de redução no país representou apenas 48% da observada na TMF na América Latina e Caribe, que foi de 1,78% ao ano. Ao comparar no âmbito global, em que o progresso na redução da mortalidade fetal nas últimas duas décadas foi de diminuição de 35% e taxa anual global de redução de 2,0%, a taxa de redução anual da TMF no país foi menor que 5%^9^.

Esses avanços na redução da TMF podem ser atribuídos às melhorias na assistência pré-natal, no atendimento ao parto e nas condições gerais de vida da população^43^. A melhoria das tecnológicas médicas permitiu maior viabilidade fetal, consecutivamente, maiores taxas de sobrevivência fetal, o que levou várias organizações internacionais a recomendarem registrar os óbitos fetais com 20ª semana ou mais de gestação, no entanto, padronizou-se, visando comparações dentro dos países, definir natimortos na IG ≥ 22 semanas de gestação e/ou 500 g ou 25 centímetros para a contagem^44,45^.

No Brasil, é normatizado para a classificação do óbito fetal a utilização do critério de 22 semanas ou mais de gestação e/ou 500 g ou 25 cm, parâmetro recomendado pela OMS adotado na CID-10, e é utilizado também no país para definir a notificação compulsória e investigação obrigatória do óbito fetal^13^. No entanto, mesmo que esse critério defina viabilidade fetal em relação a tecnologia médica disponível^18^, o país utiliza o critério de 20 semanas ou mais de gestação na obrigatoriedade da emissão da DO e do registro do óbito fetal no SIM^18^.

No contexto da viabilidade fetal, quanto mais prematuro foi o feto, maior foi o risco de morte fetal e menor a redução da mortalidade fetal no Brasil^46^. As gestações mais longas e próximas do termo apresentaram menores taxas e maiores reduções^47^. As causas prematuridade e baixo peso representaram as principais causas de mortes fetais, concomitantemente a 73% do total de natimortos no Brasil, entre 1996 e 2021. Essas mortes fetais, no período analisado, em prematuros aumentaram 2,41%, e no baixo peso ao nascer, 4,48%, dados que corroboram estudos prévios^29^.

A grande maioria (84%) dos natimortos prematuros apresentou baixo peso ao nascer (< 2.500 g). Entre os natimortos a termo, mais de um terço (37%) apresentou baixo peso. Esse dado indica que o baixo peso é um fator de risco significativo para morte fetal, mesmo em gestações a termo^48^. Apenas uma pequena minoria dos natimortos (1%), tanto pré-termo quanto a termo, apresentou peso elevado (macrossomia), sugerindo que o excesso de peso não é um fator de risco tão importante quanto o baixo peso para a morte fetal^46^.

É fundamental definir estratégias para reduzir as mortes fetais evitáveis, com atenção na prevenção da prematuridade e baixo peso ao nascer^49,50^, por meio de assistência adequada na gestação e no parto^51^. Conceptos prematuros e de baixo peso exercem grande impacto no aumento das TMF e infantil. As estratégias para reduzir a prematuridade e o baixo peso ao nascer podem ter um impacto significativo na redução dessas mortalidades^52^.

As principais causas de mortes fetais devem ser o foco de políticas e ações em saúde para a redução dessas TMF e da razão de mortalidade materna e mortalidade infantil^52^. As intervenções recomendadas antes e durante a gestação para auxiliar na redução do risco de prematuridade e baixo peso^51^ devem abordar as principais falhas no controle dessas complicações perinatais, que estão relacionadas à qualidade da assistência pré-natal, ao diagnóstico de alterações na gestacionais e ao manejo obstétrico^53^.

A classificação de natimortos pelo momento da morte em relação ao parto foi outro indicador que apontou para a necessidade de definição de intervenções específicas focadas nas diferentes fases do ciclo gravídico, principalmente durante a gravidez, para reduzir a mortalidade fetal. A maioria dos natimortos (93%) ocorreram antes do parto e uma pequena parte durante (6%). Outrossim, os natimortos anteparto aumentaram 22% e os durante diminuíram 74,52%.

Essas evidências apontam para a necessidade de investimentos em ações que qualifiquem a assistência pré-natal, para reduzir as mortes antes do parto, e em melhorias na assistência ao parto, para diminuir os óbitos durante gravidez e nascimento^54^. Por outro lado, o aumento expressivo das mortes anteparto indica falhas na assistência que precisam ser corrigidas, a fim de ampliar as chances de sobrevivência e reduzir mais as mortes fetais evitáveis^55^.

Outros estudos apontam que a morte fetal é a condição adversa mais comum na gravidez, no Brasil e no mundo, sendo determinada por múltiplos fatores. Destacam-se os fatores obstétricos, relacionados à saúde materna durante a gestação e parto; os socioeconômicos, como renda, condições de vida e alimentação da gestante; os ambientais, como exposição a riscos durante a gravidez^44^; os sociodemográficos, como idade, escolaridade e outros dados maternos^58^; e os biológicos, como condições prévias de saúde e genética da mãe e do feto^29^.

A pandemia de covid-19 intensificou esses múltiplos fatores associados às complicações fetais^15^, aumentando as mortes e freando a tendência de queda, ao acentuar vulnerabilidades preexistentes no sistema de saúde brasileiro^151^. Durante a pandemia de covid-19 (2020–2021), os aumentos cumulativos nas TMF, foram: 7,03% no biênio para ≥ 20 semanas; 6,16% para ≥ 22 semanas; e 6,12% para ≥ 28 semanas, podendo estar relacionados a fatores como o impacto da pandemia no acesso e qualidade dos serviços de saúde, além de possíveis efeitos diretos da infecção por covid-19 em gestantes^59^.

A análise das disparidades e dos grupos de risco identificados ao estratificar as TMF no Brasil, segundo características sociodemográficas e obstétricas, corrobora a influência desses diversos fatores^49^. Nesse sentido, as disparidades observadas nas TMF no país exemplificam como os múltiplos determinantes interagem de modo complexo e exercem influência na ocorrência e padrões de tendência do óbito fetal no país^44,48^. Esses dados revelam a necessidade de melhorar a qualidade da assistência obstétrica e pré-natal, bem como as condições econômicas e ambientais que afetam gestantes e fetos^12^.

Ao considerar esses múltiplos fatores na mortalidade fetal na IG ≥ 22 e ≥ 28 semanas, observam-se TMF mais elevadas entre conceptos do sexo masculino, evidenciando a influência de fatores biológicos^60^. Taxas superiores encontradas nos extremos etários maternos, como em adolescentes de 10 a 14 anos e mulheres com idade ≥ 45 anos, refletem o impacto de fatores sociodemográficos^61^. As TMF mais altas entre mulheres sem escolaridade são indicadores de desvantagem socioeconômica^62^. Partos domiciliares, gestações múltiplas, bem como partos vaginais, exibem TMF mais elevadas, ilustrando a relevância de fatores obstétricos^44^.

Os grupos de risco que comprometem a redução da mortalidade fetal, considerando a ≥ 22 e ≥ 28 semanas, incluíram: mães adolescentes (10 a 19 anos) e de idade avançada (45 a 49 anos) com a menor taxa de redução anual, mulheres sem escolaridade e com baixa escolaridade (um a três anos) com taxa anual de aumento, partos em estabelecimentos de saúde não hospitalares e domiciliares com taxa de aumento anual, gestações prematuras (inferiores a 32 semanas, principalmente aquelas com menos de 28 semanas), e feto com baixo peso (inferior a 2.500 g, especialmente inferior a 1.000 g), com menor taxa de redução anual e taxa de aumento anual.

Os grupos compostos por adolescentes muito jovens, mulheres sem instrução formal e partos domiciliares refletem desigualdades sociais, estando geralmente associados à maior vulnerabilidade social e ao menor acesso a cuidados pré-natais adequados e assistência qualificada durante o parto^63^. Idades maternas extremas (adolescentes e mulheres acima de 45 anos), assim como gestações múltiplas (gemelares e trigemelares), prematuridade e baixo peso, representam fatores de risco biológicos que aumentam as chances de complicações na gravidez, resultando em taxas mais altas de mortalidade fetal e materna^64,65^.

Para solucionar os problemas enfrentados pelos grupos de risco identificados, algumas ações específicas podem ser implementadas: ampliação do acesso a serviços de planejamento familiar e educação sexual abrangente, especialmente para adolescentes, visando prevenir gestações precoces e não planejada^66^; fortalecimento dos programas de acompanhamento pré-natal, garantindo consultas regulares, exames e orientações adequadas para todas as gestantes, com atenção especial aos grupos de risco^67^; promoção de parcerias intersetoriais e a integração de serviços de saúde, educação e assistência social para abordar os determinantes sociais subjacentes à mortalidade fetal e materna^68^.

As ações específicas devem promover a conscientização e a educação em saúde reprodutiva, enfatizando a importância do parto institucional e dos cuidados pré-natais adequados, particularmente para mulheres sem escolaridade ou com baixa escolaridade^67^. É crucial melhorar a infraestrutura e a capacitação dos profissionais de saúde em estabelecimentos de saúde, principalmente em áreas remotas ou desfavorecidas, para oferecer atendimento obstétrico de qualidade^68^. Ademais, implementar políticas de apoio social e econômico para gestantes vulneráveis, como programas de transferência de renda e assistência alimentar, a fim de melhorar suas condições de vida e nutrição durante a gravidez^68,69^.

A implementação coordenada e abrangente dessas ações pode contribuir para reduzir riscos e melhorar os desfechos para grupos vulneráveis, mitigando desigualdades e promovendo a equidade em saúde materno-infantil^68^. Negligenciar os grupos de maior risco tende a diminuir o impacto das iniciativas para redução da mortalidade fetal e perpetuar as desigualdades na mortalidade fetal^67^. Desse modo, as mortes fetais representam um significativo desafio de saúde pública, que precisa ser enfrentado para reduzir ainda mais as TMF no país^70^.

Enquanto reflexo desses desafios significativos nas condições de saúde, assistência obstétrica e determinantes sociais e econômicos que precisam ser enfrentados por meio de ações intersetoriais e políticas públicas específicas para melhorar os indicadores, a maioria das mortes fetais está vinculada a causas preveníveis, como: prematuridade, baixo peso ao nascer, complicações na gestação e parto, e fatores socioeconômicos. Potencialmente, a maioria das mortes fetais no Brasil podem relacionar-se à baixa qualidade nos cuidados prestados durante a gestação e parto^71^.

Nesse contexto, o progresso na redução dos índices de mortalidade fetal no Brasil, entre 1996 e 2021, foi influenciado por diferentes fatores e causas relacionados à gestação e ao parto, os quais ainda representam alto risco de morte fetal e perinatal^12,72^, associadas principalmente a afecções perinatais que representam 94% das causas de mortes fetais destacando-se fatores maternos, complicações da gravidez, hipóxia intrauterina e asfixia ao nascer no período analisado. Malformações congênitas foram responsáveis por 5% dos casos.

Considerada a lista de mortes por causas evitáveis, aproximadamente 67% das mortes fetais eram potencialmente evitáveis, sendo 39% reduzíveis com melhorias na Atenção Primária à Saúde e 28% evitáveis com assistência hospitalar qualificada durante o parto^73^. Esses dados refletem a necessidade de investimentos em esforços contínuos para melhorar a assistência pré-natal e ao parto, visando prevenir natimortos potencialmente evitáveis e dar continuidade ao progresso na sobrevivência fetal e infantil^7,10^.

Os natimortos são frequentemente negligenciados em discussões, planejamentos e sistemas de saúde^10^. Essa negligência fica evidente na comparação do progresso na redução dos natimortos com o dos óbitos neonatais precoces, onde houve mais sucesso (redução de 48%). Os natimortos estão em grande parte ausentes no monitoramento de dados globais^74^. Além disso, em diversos países, não se produz dados de qualidade sobre eles regularmente, dificultando a identificação das suas causas^31^.

O quadro da mortalidade fetal no Brasil, além de refletir essa negligência, parece ser um reflexo do foco na melhoria da qualidade da atenção às crianças menores de cinco anos e às mulheres, do foco no fortalecimento da obstetrícia^75^, expansão de serviços de qualidade para recém-nascidos pequenos e doentes^78^, e redução das desigualdades^79^. Porém, ao passo que essas intervenções contribuíram para maior diminuição do risco de morte neonatal, infantil e materna, o que melhorou a saúde das crianças menores de cinco anos de idade e de mulheres, e também contribuiu para a redução da mortalidade fetal e equidade no acesso à saúde materno-infantil, não teve impacto desejado sobre a saúde perinatal^80^.

Pode ser observado em âmbito mundial que a redução da TMF, na IG ≥ 28 semanas, foi de 2,0% entre 2000 e 2021, também ligeiramente superior à metade da taxa anual de redução de 3,9% na mortalidade infantil no grupo etário de um a 59 meses no mesmo intervalo de tempo^9^. Esses dados evidenciam a necessidade de esforços adicionais para acelerar a redução da mortalidade fetal, visando alcançar declínios mais próximos aos observados na mortalidade infantil^7^.

É perceptível que a mortalidade fetal não recebeu a devida atenção nas principais iniciativas globais de saúde até recentemente, quando passou a ser abordada de forma mais enfática pela OMS^7^. Em contraste à mortalidade infantil e materna, a mortalidade fetal não foi incorporada nas principais campanhas globais de saúde até 2014. Não foi incluída como indicador nas Metas de Desenvolvimento do Milênio (ODM) e Metas de Desenvolvimento Sustentável (ODS)^81^. A OMS somente intensificou o combate às mortes evitáveis, incluindo a fetal, a partir de 2014, por meio do Enap^82^ e da Estratégia Global para a Saúde de Mulheres, Crianças e Adolescentes (2016–2030)^83^.

Esse cenário global revela que a mortalidade fetal tem sido subestimada nas políticas de saúde materno-infantil^11,49^. Em âmbito global, existem lacunas significativas de informações relacionadas à mortalidade fetal, o que dificulta a compreensão completa do problema e a formulação de políticas e intervenções^29,30^. Especificamente, as políticas e os investimentos têm se concentrado principalmente em intervenções e reforço nos cuidados durante o nascimento e a primeira semana de vida do recém-nascido, deixando a fase fetal negligenciada^75^.

No entanto, apesar de ser negligenciada e dos possíveis impactos da pandemia na atenção à saúde materno-infantil, a contínua redução da mortalidade fetal no Brasil e no mundo é um resultado positivo^31^. Essas evidências indicam que as políticas públicas de saúde materno-infantil permanecem eficazes, mesmo sem focar diretamente na redução da mortalidade fetal, impactaram de forma positiva^84^. Os ganhos na diminuição dos índices desse indicador antes da pandemia, entre 1996 e 2019, foram suficientes para atenuar o aumento das taxas durante a pandemia, em 2020 e 2021.

A tendência decrescente de longo prazo das TMF não foi interrompida pela pandemia de covid-19, embora tenha ocorrido uma desaceleração na redução em comparação ao período pré-pandêmico. Com a manutenção da tendência decrescente das TMF, com apenas uma desaceleração no intervalo da pandemia de covid-19, pode-se inferir avanço na atenção materno-infantil integral^85,86^ e destacar possível reflexo da melhoria da qualidade da atenção materna e infantil, com foco no fortalecimento da obstetrícia e do cuidado perinatal, na expansão de serviços de qualidade e redução das desigualdades^78^.

As diversas intervenções contribuíram para diminuir a mortalidade infantil e materna quanto fetal, melhorando a saúde de mulheres, de crianças e promovendo equidade no acesso à saúde materno-infantil^80^. No entanto, cabe ressaltar, as evidências apontam para a necessidade de maior atenção e investimentos, com foco em políticas públicas abrangentes, coleta de dados e análise de dados adequados, e intervenções direcionadas à saúde e ao bem-estar das gestantes e fetos, incluindo crianças menores de cinco anos^12^.

Destaca-se a melhora no preenchimento das informações sobre IG e peso ao nascer no registro de natimortos, embora ainda não tenham atingido níveis plenamente adequados^38^. Em 2021, a porcentagem de dados ignorados sobre IG foi aproximadamente de 7% para natimortos, enquanto os dados ignorados sobre peso ao nascer corresponderam a cerca de 6%.

Percebe-se a necessidade de fortalecer os sistemas de informação em saúde para monitoramento e investigação das mortes fetais evitáveis^87^. A investigação dos óbitos fetais, iniciada no Brasil em 2006, reduziu em 62% os óbitos não investigados no período de 1996 a 2021. No mesmo período, houve uma diminuição de 80% nos óbitos investigados sem ficha síntese informada, e aumentou de 78% nos óbitos investigado com ficha síntese informada.

Essas evidências revelam que é crucial investir em políticas públicas de planejamento reprodutivo, pré-natal adequado e cuidados especializados para gestantes de risco, prematuros e baixo peso^88^, e ampliar o acesso e a qualidade da assistência obstétrica, incluindo o diagnóstico precoce de complicações e atendimento de emergência^31^. Especialmente, devem ser feitos investimentos em tecnologias para o reconhecimento precoce de gravidez de risco e acesso oportuno a serviços de saúde regionalizados com assistência qualificada^89^.

Por outro lado, a redução da mortalidade fetal exige maiores esforços para aumentar a conscientização pública, aprimorar a coleta de dados, avaliar o progresso e compreender as prioridades locais de saúde pública^51^. Desse modo, a chave para reduzir os natimortos e conferir-lhes visibilidade, está vinculada a investimentos em sistemas de informação em saúde e em pesquisas que gerem evidências para o aperfeiçoamento de políticas e práticas de atenção à saúde da mulher e da criança, principalmente no âmbito da saúde perinatal^12^.

O objetivo é reduzir a mortalidade fetal e mitigar os impactos dos fatores que impactam a mortalidade fetal na mortalidade infantil, incluindo a materna. Evidenciou-se que, embora a tendência decrescente de longo prazo tenha sido desacelerada durante a pandemia de covid-19, os investimentos contínuos em políticas e ações voltadas à prevenção, assistência pré-natal e parto, bem como a redução das desigualdades socioeconômicas e melhoria do acesso à educação, são fundamentais para enfrentar esse desafio e promover a equidade na saúde

### Diferenças Regionais

As evidências por Grande Região destacam desigualdades regionais na mortalidade fetal no Brasil, entre 1996 e 2021. Observam-se diferenças significativas entre as regiões, tanto no número absoluto de natimortos quanto nas TMF por 1.000 nascimentos totais. As regiões Sudeste e Nordeste concentraram o maior número absoluto de natimortos, 39% e 29%, respectivamente, provavelmente devido à maior população e número de nascimentos. No entanto, as regiões Norte (10,9/1.000) e Nordeste (12/1.000) apresentaram as maiores TMF, sugerindo maior risco de morte fetal nessas localidades.

As maiores TMF nas regiões Norte e Nordeste, com piores índices socioeconômicos^90^ e menor acesso a cuidados pré-natais e ao parto^88^, podem indicar desigualdades no acesso e qualidade dos serviços de saúde materno-infantil, bem como desigualdades socioeconômicas^58^. Essas evidências reforçam a necessidade de abordar as disparidades regionais, visando reduzir as desigualdades na mortalidade fetal em todo o território nacional^91^.

As regiões Sul, Sudeste e Centro-Oeste, presumivelmente com melhores condições socioeconômicos e maior acesso aos serviços de saúde, apresentam TMF mais baixas em comparação às regiões Norte e Nordeste. Essas evidências de diferenças regionais reforçam os achados de que a mortalidade fetal é influenciada por fatores demográficos, socioeconômicos, acesso a serviços de saúde, educação e condições gerais de vida^43^.

Padronizadas, as TMF médias anuais do Norte (10,4 ± 0,9), Nordeste (10,6 ± 1,0) e Sudeste (10,5 ± 1,9) foram semelhantes à média nacional (10,7 ± 1,0) no Brasil, e maiores que o Sul (8,6 ± 1,6) e Centro-Oeste (9,7 ± 0,9). Esses dados apontam que, enquanto algumas regiões melhoraram, outras enfrentam desafios substanciais na redução da mortalidade fetal, mesmo excluindo os fatores populacionais, reforçando desigualdades sociais e regionais persistentes^5^.

Esse cenário de disparidades regionais da mortalidade fetal no Brasil, mesmo após levar em conta diferenças populacionais, demanda mais investigações para entender os fatores por trás dessas desigualdades, como a qualidade do pré-natal, as condições dos partos e o papel dos sistemas público e privado de saúde em cada região^5^. É fundamental direcionar esforços de prevenção e melhoria para as áreas de maior risco, garantindo acesso equitativo e assistência de qualidade durante a gestação e no momento do parto, visando reduzir os índices^64^.

Não se observou expressão na redução no número de natimortos e na TMF nas Grandes Regiões brasileiras durante o período analisado, com tendência decrescente não expressiva ou estabilidade do risco de mortalidade fetal e flutuações pontuais. A região Centro-Oeste apresentou a situação mais preocupante, com quadro estável consistente da TMF nos últimos anos.

A tendência decrescente não expressiva, principalmente nas regiões Norte e Nordeste, e a estabilidade no Centro-Oeste podem ser atribuídas aos desafios persistentes nos indicadores sociais, econômicos e deficiências no acesso e qualidade dos cuidados de saúde pré-natais e parto^5^. Apesar dos esforços para implementar sistema de saúde unificado focado na atenção primária, as melhorias ainda não se refletiram significativamente na redução da mortalidade fetal^86^.

Um decréscimo mais acentuado nas TMF foi observado nas regiões Sudeste e Sul, em comparação às regiões Norte e Nordeste. Na região Sudeste, a redução nas TMF foi cerca de duas vezes maior do que na região Norte e 18 vezes mais elevada do que na região Nordeste. Similarmente, na região Sul, a diminuição nas TMF foi aproximadamente duas vezes maior do que na região Norte e 13 vezes acima da região Nordeste. Existem desigualdades regionais expressivas na mortalidade fetal no Brasil.

Quanto à mortalidade fetal, no período que inclui os anos da pandemia de covid-19 (1996–2021), a região Nordeste apresentou tendência decrescente de longo prazo. Antes da pandemia (1996–2019), essa região registrou estabilidade. A região Centro-Oeste, por outro lado, exibiu tendência decrescente antes da pandemia e estabilidade no período que abrange os anos pandêmicos, oposto ao observado no Nordeste. Nas regiões Norte, Sudeste e Sul, durante o período pandêmico, houve desaceleração na redução da mortalidade fetal, em comparação ao período anterior.

Essas tendências no período que inclui a pandemia podem ser atribuídas à intensificação de fatores como desigualdades socioeconômicas e capacidade de resposta dos sistemas de saúde locais à pandemia, bem como às possíveis diferenças nas políticas de enfrentamento à covid-19 adotadas pelos estados e municípios^15,33^. É fundamental abordar essas desigualdades para garantir assistência equitativa e de qualidade durante a gestação e o parto, considerando as especificidades e necessidades de cada região^7^.

É fundamental investir em diversas frentes para enfrentar os desafios relacionados à mortalidade fetal. Primeiramente, é necessário melhorar a atenção primária e os serviços de pré-natal, com ênfase nas regiões mais vulneráveis^11^. Além disso, é crucial fortalecer políticas que reduzam as desigualdades socioeconômicas e promovam o acesso equitativo aos serviços de saúde^48^. Outro aspecto fundamental é implementar programas de capacitação e qualificação dos profissionais envolvidos no cuidado materno-infantil, garantindo assistência de qualidade^49^.

Paralelamente, é imprescindível promover ações de educação em saúde e conscientização sobre os fatores de risco associados à mortalidade fetal, envolvendo a população^92^. Por fim, faz-se necessário aprimorar os sistemas de vigilância e monitoramento da saúde materna e fetal, a fim de subsidiar intervenções mais efetivas e direcionadas às necessidades específicas de cada região^93^. Essas medidas integradas e abrangentes são essenciais para enfrentar esse grave problema de saúde pública.

A mortalidade fetal continua sendo um dos desfechos adversos mais frequentes na gestação no Brasil, persistindo disparidades entre regiões. A falta de atenção e urgência para resolver o problema dos natimortos acarreta consequências: o progresso na sua redução torna-se lento, e o fardo permanece elevado, prejudicando a sobrevivência fetal e neonatal. Negligenciar os natimortos compromete os esforços para diminuir as mortes evitáveis de fetos e recém-nascidos. Assim, o monitoramento das mortes fetais precoces, principalmente das evitáveis, requer maior atenção e investimento, sendo fundamental para o processo de prevenção e controle da mortalidade fetal e perinatal.

## CONCLUSÃO

A mortalidade fetal no Brasil, tanto em âmbito nacional quanto nas Grandes Regiões, apresentou tendências decrescentes significativas de longo prazo durante o período pré-pandemia de covid-19 (1996–2019). Ao incluir os anos de 2020 e 2021, correspondentes à pandemia (1996–2021), essa tendência decrescente se manteve, com exceção da região Centro-Oeste, onde a tendência foi decrescente antes da pandemia e estável ao incluí-la, e da região Nordeste, que apresentou estabilidade no período pré-pandêmico e tendência decrescente de longo prazo no período total analisado.

Especificamente, a pandemia de covid-19 impactou no aumento das TMF e desacelerou a redução nos anos de 2020 e 2021. A tendência decrescente de longo prazo não foi interrompida, exceto na região Centro-Oeste. Nas Grandes Regiões, a heterogeneidade na VPA, VPMA e as disparidades das TMF refletiram desigualdades regionais.
